# DHX30 Coordinates Cytoplasmic Translation and Mitochondrial Function Contributing to Cancer Cell Survival

**DOI:** 10.3390/cancers13174412

**Published:** 2021-08-31

**Authors:** Bartolomeo Bosco, Annalisa Rossi, Dario Rizzotto, Meriem Hadjer Hamadou, Alessandra Bisio, Sebastiano Giorgetta, Alicia Perzolli, Francesco Bonollo, Angeline Gaucherot, Frédéric Catez, Jean-Jacques Diaz, Erik Dassi, Alberto Inga

**Affiliations:** 1 Department of Cellular, Computational and Integrative Biology, CIBIO, University of Trento, Via Sommarive 9, 38123 Trento, Italy; bartolomeo.bosco@unitn.it (B.B.); annalisa.rossi-1@unitn.it (A.R.); DRizzotto@cemm.oeaw.ac.at (D.R.); meriemhadjer.hamadou@unitn.it (M.H.H.); alessandra.bisio@unitn.it (A.B.); sebastiano.giorgetta@alumni.unitn.it (S.G.); alicia.perzolli@alumni.unitn.it (A.P.); francesco.bonollo@dbmr.unibe.ch (F.B.); 2 Inserm U1052, CNRS UMR5286, Centre de Recherche en Cancérologie de Lyon, Université de Lyon 1, Centre Léon Bérard, F-69008 Lyon, France; Angeline.GAUCHEROT@lyon.unicancer.fr (A.G.); frederic.catez@lyon.unicancer.fr (F.C.); JeanJacques.DIAZ@lyon.unicancer.fr (J.-J.D.)

**Keywords:** DHX30, translation efficiency, polysomal profiling, mitoribosome, ribosome biogenesis, RNA binding proteins

## Abstract

**Simple Summary:**

Translation occurs in the cell both through cytoplasmic and mitochondrial ribosomes, respectively translating mRNAs encoded by the nuclear and the mitochondrial genome. Here we found that the silencing of DHX30, an RNA-binding protein that we previously studied for its role in p53-dependent apoptosis, enhances the translation of mRNAs coding for cytoplasmic ribosomal proteins while reducing that of the mRNAs encoding for mitoribosomal proteins. This coordination of the cytoplasmic and mitochondrial translation machineries affected both cell proliferation and energy metabolism, suggesting an important role for this mechanism in determining the fitness of cancer cells. By integrating multiple datasets, we identified a gene signature that will represent a starting point to assess the prognostic value of this mechanism in cancer. We thus propose DHX30 as a potential vulnerability in cancer cells that could be exploited to develop novel therapeutic strategies.

**Abstract:**

DHX30 was recently implicated in the translation control of mRNAs involved in p53-dependent apoptosis. Here, we show that DHX30 exhibits a more general function by integrating the activities of its cytoplasmic isoform and of the more abundant mitochondrial one. The depletion of both DHX30 isoforms in HCT116 cells leads to constitutive changes in polysome-associated mRNAs, enhancing the translation of mRNAs coding for cytoplasmic ribosomal proteins while reducing the translational efficiency of the nuclear-encoded mitoribosome mRNAs. Furthermore, the depletion of both DHX30 isoforms leads to higher global translation but slower proliferation and lower mitochondrial energy metabolism. Isoform-specific silencing supports a role for cytoplasmic DHX30 in modulating global translation. The impact on translation and proliferation was confirmed in U2OS and MCF7 cells. Exploiting RIP, eCLIP, and gene expression data, we identified fourteen mitoribosome transcripts we propose as direct DHX30 targets that can be used to explore the prognostic value of this mechanism in cancer. We propose that DHX30 contributes to cell homeostasis by coordinating ribosome biogenesis, global translation, and mitochondrial metabolism. Targeting DHX30 could, thus, expose a vulnerability in cancer cells.

## 1. Introduction

The DHX30 RNA binding protein (RBP) is an ATP-dependent RNA helicase highly expressed in neural cells and somites during embryogenesis in mice. It plays an important role in development and its homozygous deletion is embryonic lethal [1]. In humans, DHX30 regulates the antiviral function of the zinc-finger ZAP protein. Indeed, it was demonstrated that these two proteins directly interact via the N-terminal domain and that DHX30 is necessary for ZAP antiviral activity [2]. Recently, de novo missense mutations were identified in DHX30 in twelve unrelated patients affected by global developmental delay (GDD), intellectual disability (ID), severe speech impairment, and gait abnormalities [3,4]. Those mutations caused amino acid changes in the highly conserved helicase motif, impairing the protein’s ATPase activity or RNA recognition. Moreover, the overexpression of DHX30 mutants led to an increased propensity to trigger stress granules (SG) formation and decreased global translation [3]. Using human fibroblasts and osteosarcoma cells, it was shown that DHX30 could also play an important role in the mitochondria. Indeed, DHX30 can interact with Fas-activated serine-threonine kinase FASTKD2, modulating mitochondrial ribosome maturation and assembly [5]. Our group and collaborators recently compared p53-mediated responses to Nutlin treatment in three different cancer cell lines: HCT116, SJSA1, and MCF7 [6,7]. It was observed that p53 activation caused cell cycle arrest in HCT116, massive apoptosis in SJSA1, and an intermediate phenotype in MCF7, consistent with earlier reports [8]. By analyzing the cell lines’ translatomes (i.e., transcripts loaded on polysomes and in active translation), we discovered that only 0.2% of the genes were commonly differentially expressed (DEGs). Furthermore, the polysome profiling of SJSA1 revealed an enrichment of pro-apoptotic transcripts in the fraction corresponding to polysomes [7]. Those transcripts featured instances of a specific cis-element in their 3′UTR that we labeled CGPD, standing for CG-rich motif for p53-dependent death. The DExH box RNA helicase DHX30 was found to be one RBP whose binding to the CGPD motif correlated with cell fate. DHX30 was expressed at higher levels in HCT116 than in SJSA1 and associated with the lower translation of GCPD-motif-containing transcripts in HCT116 cells. Indeed, the depletion of DHX30 in HCT116 by stable shRNA increased the translation potential of mRNAs containing the CGPD motif, resulting in higher levels of apoptosis after p53 activation by Nutlin treatment, mimicking in part the apoptotic phenotype of SJSA1 cells [7]. In this work, we set to determine the broader phenotypic role of DHX30 in the colorectal cancer cell line HCT116. Two alternative DHX30 promoters led to the production of isoforms containing or not containing a mitochondrial localization signal. Indeed, we show that DHX30 is present and active both in the mitochondria and in the cytosol and that it can couple mitochondrial function, ribosome biogenesis, and global translation. Depleting DHX30 in HCT116 cells enhanced global translation but reduced cell proliferation, as also confirmed in two other cancer cell line models.

## 2. Materials and Methods

### 2.1. RNA-seq Library Preparation and Sequencing

HCT116_shDHX30 and HCT116_shNT control cells were kept untreated and processed 12 h after seeding. Polysomal profiling and RNA extraction of reconstituted total cytoplasmic fractions was performed as recently described [7]. Sequencing libraries were obtained following the manufacturer instructions of the TruSeq RNA Library preparation kit v2. We used 1.5 µg of RNA as input and assessed the input RNA quality by the Agilent RNA 6000 Nano kit on an Agilent 2100 Bioanalyzer instrument. Four replicates were generated for each condition. The resulting 16 samples were sequenced on a HiSeq 2500 machine producing ~25M raw reads per each sample.

### 2.2. RNA-seq Data Analysis

Reads were first quality filtered and trimmed with trimmomatic (minimum quality 30, minimum length 36nt) [9]. Then, each Gencode v27 (http://www.gencodegenes.org/releases/, accessed on 10 October 2017) transcript was quantified with Salmon [10]. Eventually, edgeR [11] was used to call Differentially Expressed Genes (DEGs) between conditions at the polysomal and total levels (shDHX30 DMSO vs. shNT DMSO) using a 0.05 threshold on the adjusted *p*-value. GSEA was performed with the fgsea R package [12], including the Hallmark, Canonical Pathways, and GO gene sets. We used 1000 permutations to compute the significance *p*-value and used a BH adjusted *p*-value threshold of 0.05. Translational efficiency (TE) was computed on normalized expression data (counts per million reads, CPM) as the ratio of polysomal CPMs over total CPMs for each replicate and transcript in the annotation. Differences in TE (between conditions and groups of genes) and between polysomal and total samples were assessed by the Wilcoxon test. Motifs in ribosomal and mitoribosomal genes 5′- and 3′-UTRs were obtained with DREME [13] using a 1 × 10^−4^ *p*-value threshold and shuffled input sequences as controls.

### 2.3. Cell Lines, shRNAs, and Culture Conditions

HCT116 and U2OS shNT control or shDHX30 clones were obtained as recently described [7]. Briefly, cells were transduced with lentiviral vectors containing a pLK0.1 plasmid expressing shRNAs. DHX30 targeting sequences are: TRCN0000052028 (GCACACAAATGGACCGAAGAA, targeting the common exon ENSE00003493567), TRCN0000052031 (CCGATGGCTGACGTATTTCAT, targeting exon ENSE00003510564), and TRCN0000052032 (GAGTTGTTTGACGCAGCCAAA, targeting exon ENSE00003669522). All three shRNAs target DHX30 exons that are common to all annotated coding transcripts for the gene. Stable clones were selected, exploiting the puromycin resistance marker. Single clone isolates were obtained and characterized for DHX30 protein depletion. For the HCT116 cell line, most experiments were performed using one single-cell-derived clone expressing the shRNA28. Consistent results were obtained with single-cell clones expressing shRNA31 (clones A8 and G3) and shRNA32 (clones A9 and C10) (Figures S1 and S2). MCF7 cells were purchased from ICLC (IRCCS San Martino Hospital, Genoa, Italy). All cell lines were cultured in RPMI (Gibco, Thermo Fisher Scientific, Waltham, MA, USA) supplemented with 10% Fetal Bovine Serum (Gibco, Thermo Fisher Scientific) and 1X L-Glutamine and Pen/Strep (Gibco, Thermo Fisher Scientific) at 37 °C with 5% CO_2_ in a humidified atmosphere. U2OS cells were cultured in DMEM pH 7.4 (Gibco, Thermo Fisher Scientific) supplemented with 10% Fetal Bovine Serum (Gibco, Thermo Fisher Scientific) and 1X L-Glutamine and Pen/Strep (Gibco, Thermo Fisher Scientific) at 37 °C with 5% CO_2_ in a humidified atmosphere. For the routine culture of depleted clones and controls, 0.2 µg/mL Puromycin was added to the culture medium to maintain the selection of the vectors. Puromycin was removed 24 h before starting a specific experiment to avoid confounding effects. MCF7, U2OS, and HCT116 parental cells were silenced for cytoplasmic, mitochondrial, or simultaneous cytoplasmic and mitochondrial DHX30 variants using 25 nM (for HCT116) or 50 nM (for MCF7 and U2OS) of three different siRNAs (Trifecta, IDT, Coralville, IA, USA) (Table S3) transfected using Interferin (Polyplus-transfection, Illkirch, France). All experiments were performed at least 24 h post-silencing.

### 2.4. Western Blot

Cells were collected with trypsin-EDTA at appropriate time points, centrifuged and washed with PBS. Samples were then lysed by RIPA buffer and the proteins were quantified by BCA assay (EUROCLONE, Milan, Italy). 30–50 µg of extracted proteins were loaded on 8%, 12%, or 15% Tris-glycine Gel and then transferred onto a nitrocellulose membrane using Tris-Glycine buffer. Blocking was performed for 1 h with 5% not-fat dry milk in PBS 1X containing 0.1% TWEEN, or with the SuperBlock blocking buffer (ThermoFischer Scientific, Waltham, MA, USA) for phosphoproteins’ detection. Immunodetection was obtained using primary and secondary antibodies reported in Table S4. The membranes were analyzed by ECL and detected by ChemiDoc XRS+ (Bio-Rad, Hercules, CA, USA) using ImageLab software (Bio-Rad).

### 2.5. Polysome Profiling

Polyribosome analysis was performed as described in [14,15,16]. Briefly, HCT116 cells were grown on 15 cm Petri dishes with standard media and serum. When the cells reached 80% of confluence, cycloheximide (0.01 mg/mL, Sigma Aldrich, St. Louis, MO, USA) was added and kept in incubation for 10 min. Then, the cells were washed two times with cold PBS containing cycloheximide (0.01 mg/mL) and lysed using the following lysis buffer: 20 mM Tris-HCl (pH 7.5), 100 mM KCl, 5 mM MgCl_2_, 0.5% Nonidet P-40, and 100 U/mL RNase inhibitors. Mitochondria and nuclei-free lysates were loaded onto 15–50% (*w*/*v*) density sucrose gradients in salt solution (100 mM NaCl, 5 mM MgCl_2_, and 20 mM Tris–HCl pH 7.5) and ultracentrifuged at 180,000× *g* for 100 min at 4 °C. The sedimentation profiles were monitored by absorbance at 254 nm using a Teledyne ISCO UA-6 fractionator coupled with a UV detector, collecting 13 fractions of 1 mL each. RNA from the pooled polysomal fraction was extracted and processed as described in [6]. For Western blot analysis, 100 uL of 100% TCA and 1 mL of ice-cold acetone were added to 1 mL of each fraction. Then, the samples were put at −80 °C overnight to induce protein precipitation. Subsequently, the samples were centrifuged at 16,000× *g* for 10 min at 4 °C and washed three times with 1 mL of ice-cold acetone. Finally, the pellets were solubilized directly in Laemmli buffer pH 8.

### 2.6. Ribosome Isolation

Ribosome isolation was performed following a protocol previously described [17]. Briefly, 8 × 10^6^ cells were seeded in a T150 flask, 48 h before the procedure. At 80% confluence, the cells were detached and counted. 1 × 10^7^ cells were pelleted by 500× *g* centrifugation for 5 min at 4 °C and washed once with cold PBS. The supernatant was discarded and the pellet was resuspended gently with 300 µL of cold buffer A (sucrose 250 mM, KCl 250mM, MgCl_2_ 5 mM, and Tris-Cl 50 mM pH 7.4) added in three sequential steps, pipetting after every addition. To perform cell lysis, an appropriate volume of NP-40 was added to the homogenized cellular solution to obtain a 0.7% (*v*/*v*) final concentration of the detergent, and cells were incubated on ice for 15 min, homogenizing the suspension by gentle pipetting every 5 min. Then, the cell lysate was centrifuged at 750× *g* for 10 min at 4 °C to pellet nuclei; the recovered cytoplasmic fraction (supernatant) was centrifuged again at 12,500× *g* for 10 min at 4 °C to obtain a mitochondria pellet. The supernatant containing ribosomes was collected and its volume was accurately measured using a graduated pipet. KCl 4 M solution was then slowly added to reach a final concentration of 0.5 M. Meanwhile, 1 mL of the sucrose cushion solution (sucrose 1M, KCl 0.5M, MgCl_2_ 5 mM, and Tris-Cl 50 mM pH 7.4) were added into a 3-mL polycarbonate tube for an ultracentrifuge TL100.3 rotor (Beckman Coulter, Brea, CA, USA). The KCl-adjusted ribosome-containing solution was carefully added above the sucrose cushion and tubes were balanced by weight using buffer B (sucrose 250mM, KCl 0.5 M, MgCl_2_ 5 mM, and Tris-Cl 50 mM pH 7.4). Then, they were ultracentrifuged at 250,000× *g* for 2 h at 4 °C. At the end of the centrifugation, the supernatant was discarded and a very compact and dense translucent pellet containing ribosomes was quickly rinsed twice by carefully adding 200 µL of cold water. Then, the pellets were resuspended in 300 µL of buffer C (KCl 25 mM, MgCl_2_ 5 mM, and Tris-Cl 50 mM pH 7.4), adding the solution in three sequential steps and gently homogenizing by pipetting after every 100 µL addition. Finally, to estimate the number of ribosomes, absorbance at 260 nm of the suspensions was measured by a nanodrop spectrophotometer (ThermoFisher).

### 2.7. Global Translation

Global translation assay was performed using Click-iT AHA Alexa Fluor 488 Protein Synthesis HCS Assay (Thermo Fisher Scientific). Briefly, HCT116 shNT and shDHX30, U2OS shNT and shDHX30, or HCT116 and MCF7 cells transiently silenced for DHX30 were cultured in 96-well tissue culture plate and treated with 100 µM 5-Fluorouracil (Sigma Aldrich) or the MTOR inhibitors Rapamycin (10 µM) and Torin (100 nM) (Sigma Aldrich)—combined to maximize the effect—as a control for reduced global translation. After 24 h of treatment, the culture medium was removed, 50 µM L-azidohomoalanine prepared in pre-warmed L-methionine-free medium (Lonza) was added, and the cells were incubated for 1 h. The cells were fixed in 3.7% formaldehyde (Sigma Aldrich), incubating the plate at room temperature for 15 min. After washing with 3% BSA in PBS 1X, the cells were permeabilized with 0.5% Triton X-100 (Sigma Aldrich) and incubated for 15 min at room temperature. The cells were washed twice with 3% BSA in PBS 1X, and Click-iT reaction cocktail was added, incubating for 30 min at room temperature and protected from light. After the incubation reaction cocktail was removed, the cells were washed twice with 3% BSA in PBS 1X and stained with 5 µg/mL Hoechst (Sigma Aldrich). The samples’ images were acquired and analyzed by a high content fluorescence microscope Operetta (PerkinElmer, Waltham, MA, USA) using the following filter set: Alexa Fluor 488: Ex495/Em519 nm; Hoechst: Ex350/Em461 nm. The average cytoplasmic AHA Alexa Fluor 488 intensity signals after background subtraction were compared.

### 2.8. Fluorescent In Situ Hybridization (FISH) on rRNA Precursors

The cells were cultured on a coverslip place onto a 48-well plate and after 48 h they were washed with PBS and fixed with 4% Paraformaldehyde (PFA) (Sigma Aldrich) for 30 min at RT. Then, the cells were washed twice with PBS 1X and incubated in 70% ethanol overnight at 4 °C. The day after, the cells were rehydrated with 10% formamide in SSC 2X (Sigma Aldrich), twice for 5 min at RT. Meanwhile, buffer A [5 µL formamide, 2.5 µL SSC 2X, 2.5 µL tRNA (10 ng/µL), water up to 20.25 µL, and 2.5 µL of each probe (10 ng/µL)] was incubated for 5 min at 90 °C and then mixed quickly together with buffer B [25 µL Dextran sulfate 20% in SSC 4X, 1.25 µL Bovine Serum Albumin (BSA) (10 ng/µL), and 2.5 µL ribonucleoside vanadyl complex (RVC) (200 mM)]. Then, 50 µL A+B probe solution was dropped onto the coverslip and was incubated in a humidified chamber for 3 h at 37 °C. The coverslips were washed twice with 10% formamide in SSC 2X for 30 min at RT and once with PBS 1X for 5 min. The cells were stained with a solution of 0.5 µg/mL DAPI (Sigma Aldrich) in PBS 1X for 5 min, washed three times with PBS 1X, and the coverslip was finally placed with a mounting medium on the glass slide. Finally, the images were acquired using a Zeiss Observer z1 fluorescent microscope (Carl Zeiss, Oberokochen, Germany) with ZEN 2 blue edition software ver. 2.3 (Carl Zeiss). The images’ fluorescence intensity was measured using Cell Profiler software ver. 3.1.9 (Broad Institute, Cambridge, MA, USA). 

### 2.9. rRNA Synthesis

rRNA synthesis was evaluated following the protocol described by [18,19]. Briefly, the cells were cultured in 96-well tissue culture plates and, after 24 h, were treated with 100 nM Actinomycin D (Sigma Aldrich) as positive control. After 24 h of treatment, 1 mM 5-Ethynyluridine (Sigma Aldrich) was added to the culture medium and the plate was incubated for 2 h. This short incubation with 5-EU allows for a higher rate of incorporation in rRNAs than mRNAs, reducing the background signal. Then, the cells were fixed using a working solution 1 composed by 125 mM Pipes pH 6.8 (Sigma Aldrich), 10mM EGTA (Sigma Aldrich), 1 mM MgCl_2_ (Merck Millipore, Burlington, MA, USA), 0.2% Triton X-100 (Sigma Aldrich), and 3.7% formaldehyde (Sigma Aldrich). The cells were washed twice with TBS 1X and stained for 30 min at room temperature using the working solution 2 composed by 100 mM Tris-HCl pH 8.5 (Sigma Aldrich), 1 mM CuSO_4_ (Merck Millipore), 10 µM fluorescent azide (Sigma Aldrich), and 100 mM ascorbic acid (Merck Millipore). After four washes with TBS containing 0.5% Triton X-100, the cells were stained with 0.5 µg/mL Hoechst (Sigma Aldrich) and the samples’ images were acquired and analyzed by a high content fluorescent microscope Operetta (PerkinElmer) using the following filter set: Alexa Fluor 488: Ex495/Em519 nm; Hoechst: Ex350/Em461 nm and setting a threshold on Alexa Fluor 488 at 500 fluorescent units to plot the percentage of cells with high nucleolar signal intensity.

### 2.10. Proteasome Activity

Proteasome activity assay was performed using the 20S Proteasome Assay Kit (Cayman Chemical). Briefly, the cells were cultured in 6-well tissue culture plates. The day after seeding, cells were washed with the 20S proteasome assay buffer, detached, and lysed with the 20S proteasome lysis buffer. 90 µL of supernatants were transferred in a black 96-well plate and 10 µL of assay buffer were added in each well. The assays utilize a specific substrate that, upon cleavage, generates a 480 nm fluorescence readout. Jurkat cell lysate and epigallocatechin gallate (EGCG), a 20S inhibitor, were used as positive and negative controls, respectively.

### 2.11. Colony Formation

1.0 × 10^3^ HCT116 shNT and shDHX30 cells per well were seeded in a 6-well plate and incubated. The culture medium was changed every three days. The colonies were fixed with 3.7% formaldehyde and then stained with a solution containing 30% Methylene blue (Sigma Aldrich) in water and washed five times for 5 min. The images were acquired and analyzed using ImageJ software 1.8.0 (National Institute of Health, Bethesda, MD, USA).

### 2.12. Cell Count by High-Content Analysis and Real Time Cell Index Analysis

To analyze cell proliferation by cell count, 1.0 × 10^3^ cells per well were seeded in 96-well plates and incubated. The images were acquired by the high content fluorescent microscope Operetta (PerkinElmer) in digital phase contrast every 24 h for three or four days. To analyze cell proliferation by Real Time Cell Analyser xCELLigence (RTCA) (Roche, Basel, Switzerland) the background was set adding the only cell culture medium in each well of the E-plate and incubating for 30 min. After incubation, the background was read. Then, 1.0 × 10^3^ cells per well were seeded and incubated for 30 min, the E-plate was inserted in the RTCA, and impedance-based cell proliferation was estimated by RTCA readings every 15 min over three days. 

### 2.13. Spheroid Assay Formation

3.0 × 10^3^ HCT116 shNT and shDHX30 cells per well were seeded in a U-bottom ultra-low attachment 96-well plate (Corning Incorporated, Corning, NY, USA), centrifuged at 3000 rpm for 5 min and incubated. Starting after three days of incubation, images were acquired every 24 h for eight additional days by the microscope (Leica, Wetzlar, Germany). The spheroids’ area was measured by ImageJ software.

### 2.14. Immunofluorescence

3.0 × 10^5^ cells per well were seeded on glass coverslip inserted in 6-well plates and incubated overnight. The days after, the cells were fixed in 4% Paraformaldehyde (PFA) for 15 min. Then, the PFA solution was removed and the cells were rinsed twice with PBS 1X. After washing, the cells were incubated for 1 h with blocking solution (PBS 1X, 5% BSA, and 0.5% Triton X-100). Next, the cells were incubated overnight at 4 °C with primary antibodies (Table S4) diluted in blocking solution. The following day, after three washes with PBS 1X, the anti-mouse and anti-rabbit secondary antibodies conjugated with AlexaFluor 488 or AlexaFluor 594 (Invitrogen, Carlsbad, CA, USA) (diluted 1:1000) were added to the samples and incubated for 1 h at RT under agitation. The cells were washed with PBS 1X three times and cell nuclei were stained with 0.5 µg/mL Hoechst (Sigma Aldrich) (diluted 1:5000). Finally, the coverslips were mounted on glass slides and the images were acquired using a Zeiss Observer Z1 fluorescent microscope and ZEN 2012 software (Carl Zeiss Jena, Germany). About 30 to 35 cells were imaged for each condition. The list of primary antibodies used is in Table S4.

### 2.15. Compensatory Glycolytic Test

3.0 × 10^3^ cells per well were seeded in an XFp cell culture microplate (Agilent Technologies, Santa Clara, CA, USA) and incubated overnight, while XF calibrant solution was added in each well of the extracellular flux cartridge and incubated overnight without CO_2_. The day after, the culture media was removed from cells, replaced with assay medium, and the microplate was incubated 45 min at 37 °C without CO_2_. The extracellular flux cartridge was prepared adding 0.5 µM rotenone/antimycin A in port A and 50 mM 2-Deoxyglucose in port B diluted in XFp assay medium, and the cartridge was calibrated by the Seahorse XFp analyzer using the setting for the Glycolysis Test program. When the calibration was finished, the cell microplate was inserted in a Seahorse XFp analyzer to start the analysis.

### 2.16. RNA Extraction and RT-qPCR

3.0 × 10^5^ cells per well were seeded in a 6-well plate and incubated overnight. The day after, the cells were starved and lysed with β-mercaptoethanol and RNA was extracted using an Illustra RNAspin Mini RNA Isolation kit (GE-Healthcare, Chicago, IL, USA) following the kit protocol. Then, 500–1000 ng of RNA were retro-transcribed using the RevertAid RT Kit (Thermo Fisher Scientific). Finally, RT-qPCR was performed using 25 ng of cDNA and 2X qPCR SyGreen (PCR Biosystem, London, UK) in Cfx96 Real-Time System or Cfx384 Real-Time System Thermocyclers (Bio-Rad). The sequence of all primers used is reported in Table S3.

### 2.17. Digital Droplet PCR

Digital droplet PCR was performed based on the protocol developed by Bio-Rad. Briefly, 1 × 10^5^ cells were seeded in a 6-well plate and grown for 1 day. Next, the cells were detached, counted, and solubilized in DireCtQuant 100ST solubilization reagent (DireCtQuant, Lleida, Spain) at the concentration of 1000 cells/µL, incubating for 3 min at 90 °C with shaking at 750 rpm and then centrifuged for 1 min at 10,000× *g*. The lysates were obtained by incubation for 3 min at 90 °C under shaking at 750 rpm and then centrifuged for 1 min at 10,000× *g*. The lysate was diluted 1:4 in the solubilization reagent, and for every target to analyze, a PCR mix was prepared comprising 2 µL of diluted sample and with forward and reverse primers at 10 µM final concentration in a final volume of 95 µL. PCR mix without lysate was used as negative control. Then, 10.5µL of each PCR reaction were mixed with 11 µL of 2xQX200 ddPCR EvaGreen SuperMix (Bio-Rad) and 0.5 µL of HindIII restriction enzyme (NEB, Ipswich, MA, USA). Sample DNA was digested at 37 °C for 15 min and loaded in the ddPCR DG8 Cartridge with QX200 droplet generation oil (Bio-Rad). The droplets were made using a QX200 droplet generator (Bio-Rad) and then loaded in a 96-well PCR plate. Sample DNA was amplified, and droplets with amplified DNA were analyzed using the QX200 droplet reader and QuantaSoft 1.7.4 (Bio-Rad).

### 2.18. RIP Analysis 

The HCT116 cells were cultured in a standard medium in P150 plates until they reached ~80% confluence. In total, ~107 cells were lysed in 1 mL of Lysis buffer (100 mM KCl, 5mM MgCl_2_, 10 mM HEPES pH 7, 0.5% NP-40, 1 mM DTT, 1 U/uL RNase Inhibitors, 1X Protease Inhibitor Cocktail) using a scraper. The lysates were transferred in a falcon tube, placed for at least two hours at −80 °C, and centrifuged at 10,000× *g* rpm for 30 min. the supernatants were collected in a new tube. Dynabeads ProteinA or ProteinG (depending on the antibody species, Thermo Fisher scientific) were prepared by washing them twice with NT2 Buffer (50 mM Tris-HCl pH 7.4, 150 mM NaCl, 1 mM MgCl_2_; 0.05% NP40) and resuspended in NT2 buffer. The beads were distributed in different tubes, supplemented with twice their initial volume of NT2 Buffer. The DHX30 specific antibody (5 µg -A302-218A, Bethyl) or IgGs were added to the beads and incubated for 2 h on a rotating wheel at 4 °C. The lysates were pre-cleared by adding a mix containing ProteinA and ProteinG Dynabeads in equal amounts and incubating for 1 h at 4 °C on a wheel. After placing the tubes on a magnet, the supernatants were collected and 1% of their volume was used as input to be directly extracted with TRIzol. The remaining supernatant was added to the antibody-coated beads and incubated overnight on a wheel at 4 °C. The day after, the beads were washed with 1 mL NT2 buffer for 10 min on a wheel at 4 °C. Three additional washes were performed again with 1 mL NT2 Buffer supplemented with 0.1% Urea + 50 mM NaCl (10 min each at 4 °C on a wheel). The beads were washed one more time in 500 µL of NT2 buffer, 50 µL were collected for WB analysis, and the remaining supernatant was discarded. RNA was extracted by adding TRIzol to the beads, according to the manufacturer’s protocol. The RNA pellets were resuspended in 15 µL DEPC water and cDNAs were synthesized using the RevertAid First Strand cDNA Synthesis Kit (Thermo Fisher Scientific).

### 2.19. Cytoplasmic-Mitochondrial Fractionation

For cytoplasmic-mitochondrial fractionation, 2 × 10^6^ cells were seeded in a P150 Petri dish and incubated for 2 days. Then, the cells were detached using a scraper and re-suspended in 1 mL of fractionation buffer (250 mM Sucrose, 10 mM Tris-HCl pH 7.5). The cells were homogenized by applying 20 strokes using a 25 G 0.5 × 16 mm syringe and 50 uL homogenate was collected as a whole cell lysate reference. A differential centrifugation was applied to separate different fractions. To remove unbroken cells, the homogenate was centrifuged for 5 min at 300× *g* at 4 °C and the supernatant was again centrifuged for 10 min at 1000× *g* at 4 °C to separate nuclei. The collected supernatant was then centrifuged for 10 min at 7000× *g* also at 4 °C to pellet the mitochondria. The remaining supernatant was collected as the cytoplasmic fraction. Finally, the mitochondrial pellet was washed once in fractionation buffer and collected by centrifugation for 10 min at 7000× *g* at 4 °C. The pellet was then lysed in RIPA buffer. All proteins’ fractions were quantified by BCA assay to be equally loaded on the acrylamide gels.

### 2.20. Apoptosis Assay

Apoptosis analysis was based on FITC Annexin-V Apoptosis Detection Kit protocol (BD Biosciences, San Jose, CA, USA). Briefly, 3 × 10^5^ cells per well were seeded in a 6-well plate, incubated for one day, and then treated with selected drugs for 48 h. 0.1% DMSO was used as negative control. The cells were detached using trypsin and washed twice with PBS 1X. Then, 1.5 × 10^5^ cells were resuspended in 100 µL of Binding Buffer 1X, stained with FITC-Annexin V and PI, and incubated for 15 min at room temperature in the dark. Finally, the samples were analyzed by FACS Canto flow cytometer and BD Diva Software 6.1.3. Unstained, PI-only, and FITC-only stained samples were used to set the cytometer’s parameters. 

### 2.21. Cell Cycle Analysis

HCT shNT or shDHX30 were seeded in 6-well plates at a concentration of 3 × 10^5^ cell/well. 48 h after seeding, the cells were trypsinized, washed with 1X PBS, and counted for subsequent staining according to BD Cycletest Plus DNA Kit protocol. Briefly, 5 × 10^5^ cells were centrifuged, the supernatant was removed, and 250 µL of Solution A were added. The cells were resuspended and incubated for 10 min before adding 200 µL of Solution B. After 10 min of incubation, 200 µL of ice-cold Solution C were added to the samples and the cells were incubated for 10 min at 4 °C in the dark before analysis with FACS CantoA (BD). The percentage of cells in each stage of the cell cycle (G1, S or G2) was computed using the ModFIT LT 4.0 software (Verity Software House). 

### 2.22. Exploration of TGCA Data Using the GEPIA Web Resource

The GEPIA API [20,21] was used to extract correlations between DHX30 and mitoribosomal genes (either as individual genes or for the 15-gene signature) in all available TCGA tumor datasets (correlation mode). The same analysis was performed to correlate cytoplasmic ribosome and mitochondrial ribosome genes expression on TCGA tumor samples, matched TCGA normal samples, and GTEX healthy tissues RNA-seq data. Survival analysis was performed with the same tool and in the same datasets, using the survival mode. Only results within the *p*-value threshold of 0.05 were considered, with the others being set to R = 0 and HR = 1 in the heatmap for visualization purposes.

### 2.23. Statistical Analysis

The data are presented as mean ± SD of three independent biological experiments, unless stated otherwise. GraphPad Prism 5 or 9 software (GraphPad software, La Jolla, CA, USA) were used, and either a one-way or two-way ANOVA with a multiple comparison test or a two-tailed Student’s *t*-test were performed, unless otherwise specified. 

## 3. Results

### 3.1. DHX30 Depletion Enhances Translation in HCT116 Cells

We previously identified DHX30 as a negative modulator of p53-dependent apoptosis, performing polysomal profiling followed by RNA-seq of HCT116 cells clones stably depleted for DHX30 and treated with the MDM2 inhibitor Nutlin [8]. Here, we reanalyzed that dataset (GSE95024), complementing it by performing a total-cytoplasmic RNA profiling of the same cells by RNA-seq (GSE154065) and focusing on the comparison between DHX30-depleted (shDHX30) cells and control (shNT) HCT116 cells in the untreated condition. shRNAs led to about 80% reduction in DHX30 protein levels (Figure S1B,C,H).

**Figure 1:**
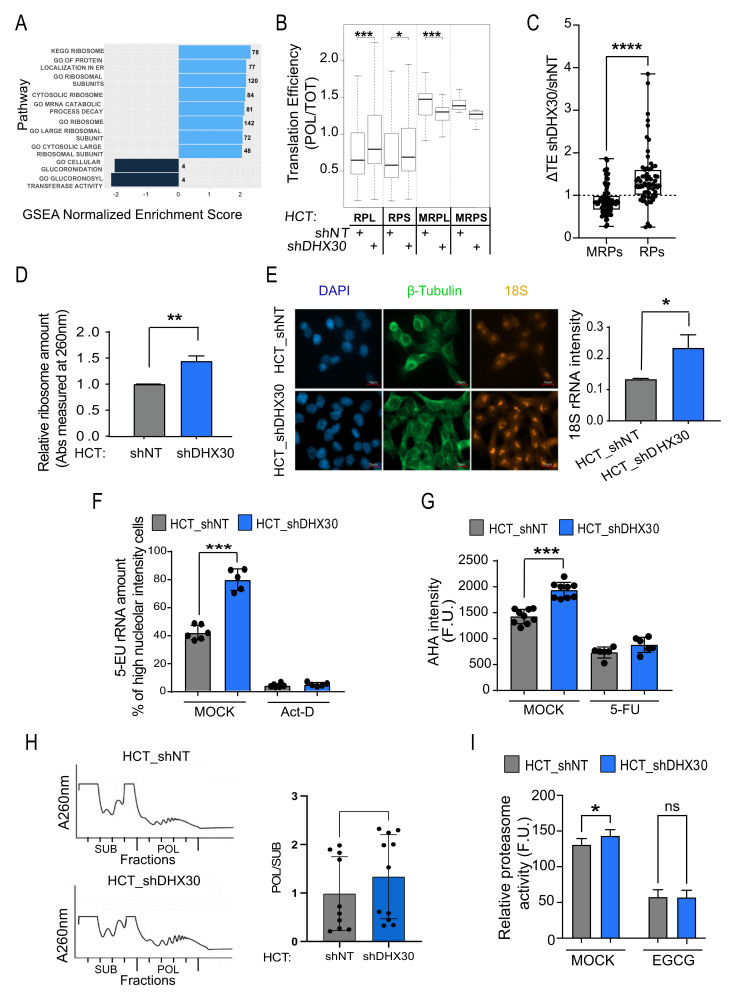
DHX30 depletion increases ribosome biogenesis and translation in HCT116 colorectal cancer cells. (**A**) Most significant gene sets, as determined by the Gene Set Enrichment Analysis of the polysome-bound HCT_shDHX30 vs. HCT_shNT differential expression (1000 permutations, adjusted *p*-value threshold = 0.05) among KEGG and REACTOME pathways, Cancer hallmarks, and Gene Ontology terms gene sets. Bars plot the Normalized Enrichment Score, (NES). Numbers indicate the number of genes in the leading edge. See Table S1 for full results. (**B**) Box plot of Translation efficiency for the indicated transcript groups. The RNA-seq CPM in polysomal over total RNA was measured. Results obtained in HCT116_shDHX30 cells are compared to the shNT control clone; * *p* < 0.05, *** *p* < 0.001, *t*-test. (**C**) Dot plot of the ratio between the translation efficiency of eight MRPs or RPs transcripts in the comparison between RT-qPCR data obtained with HCT116_shDHX30 and HCT_shNT. Each dot plots the result of one individual replicate. Polysome-associated mRNAs were extracted after sucrose-gradient fractionation. Data for the individual transcripts are presented in Figure S1A; **** *p* < 0.0001, Mann-Whitney test. (**D**) Number of ribosomes produced in HCT116_shDHX30 and HCT_shNT. After ribosomes isolation, protein absorbance was measured at λ 260 nm. Data are normalized on shNT and are mean ± SD (n = 3); ** *p* < 0.01, *t*-test. (**E**) (Left) Representative images of one of three independent experiments of Fluorescent In Situ Hybridization (FISH) for rRNA precursor 18S (red). Immunofluorescence of tubulin (green) and staining with DAPI (blue) were used to visualize cells. (Right) Box plot of 18S rRNA intensity quantification comparing HCT116_shDHX30 with HCT_shNT analyzed with Cell Profiler software. Data are expressed as mean per well ± SD of three biological replicates in which 10 nuclei were quantified for each replicate; * *p* < 0.05, *t*-test. (**F**) Amount of ribosomal RNA estimated based on the fluorescence intensity of 5-ethynyl uridine (5-EU) incorporated in nascent rRNAs present in the nucleoli. HCT116_shDHX30 cells are compared to the shNT control clone in untreated condition while Actinomycin-D treatment was used as a control. Data are mean ± SD (n = 6); *** *p* < 0.001, *t*-test. (**G**) Analysis of global translation based on the fluorescence intensity of L-azidohomoalanine (AHA) incorporated in nascent proteins present in the cytoplasm. HCT116_shDHX30 cells are compared to the shNT control clone in untreated condition, while 5-Fluorouracil treatment was used as the control of reduced global translation. Data are mean ± SD (n = 6 to 9); *** *p* < 0.001, *t*-test. (**H**) (Left) Polysome profiling of HCT_shNT and HCT_shDHX30, revealed by the measurement of absorbance at a wavelength of 260 nm. (Right) Box plot of the relative quantification of the areas under the curve of fractions corresponding to the polysomes relative to the fractions corresponding to the ribosomal subunits and 80S monosome (POL/SUB, an estimate of translation efficiency) after interpolating the data to correct for peak saturation. The mean and the individual values are shown (n = 10); * *p* < 0.05, paired *t*-test. (**I**) Relative proteasome activity expressed as fluorescence units in HCT_shNT and HCT_shDHX30 cells in mock condition or after treatment with epigallocatechin gallate (EGCG), a 20S proteasome inhibitor. Data are plotted as mean ± SD (n = 3); ns = not significant; * *p* < 0.05, *t*-test.

We compared the gene expression changes at the polysomal or total cytoplasmic RNA levels and performed a gene set enrichment analysis [12]. This revealed an increased abundance of ribosomal proteins and ribosome biogenesis genes in shDHX30 cells, only at the polysomal level (Figure 1A, Table S1). Consistently, the analysis of translation efficiency (TE), measured as the ratio between relative RNA abundance in polysomal and total cytoplasmic fractions, revealed that DHX30 depletion is associated with higher TE for transcripts coding for ribosomal protein subunits (RPL, RPS, Figure 1B). On the other end, nuclear transcripts coding for mitochondrial ribosomal proteins exhibited reduced TE in DHX30-depleted cells, despite showing higher constitutive TE compared to the cytoplasmic ribosomal transcripts (MRPL, MRPS, Figure 1B). Validation by RT-qPCR was performed for eight MRPs and eight RPs transcripts. The observed difference in TE between the grouped ribosome and mitoribosome transcripts (Figure 1C), as well as for individual mRNAs (Figure S1A), confirmed the impact of DHX30 depletion measured from the RNA-seq data. A trend for the higher expression of RPL and RPS transcripts and the lower expression of MRPL and MRPS transcripts in DHX30-depleted cells was also apparent (Figure S1D). 

Consistent with the higher TE for RPL and RPS transcripts, DHX30 depletion in HCT116 cells was associated with higher relative numbers of ribosomes extracted and quantified from a sucrose cushion (Figure 1D) and with higher rRNA levels (Figure 1E,F). Furthermore, DHX30-depleted cells not only showed evidence of increased ribosome biogenesis but also of higher global translation (Figure 1G,H) and a slight increase in proteasome activity (Figure 1I). The higher global translation did not appear to be related to an increase in MYC expression nor activity (Figure S1F) but was associated with a slight induction in the mTOR pathway (Figure S1E,H). Finally, the DHX30 protein was found to be associated with ribosomal subunits, 80S monosomes, and light polysomes based on the polysome profiling of HCT116, MCF7, and U2OS cells (Figure S1G). Consistent results for global translation and 4EBP1 phosphorylation were obtained with three different single cell-derived stably depleted clones expressing two additional shRNAs targeting DHX30 exons (Figure S1H,I). 

Collectively, we infer from the depletion experiments that DHX30 acts to negatively influence ribosome biogenesis, global translation, and also the translation of specific mRNAs.

### 3.2. DHX30 Depletion Alters the Expression of Mitoribosomal Transcripts

**Figure 2:**
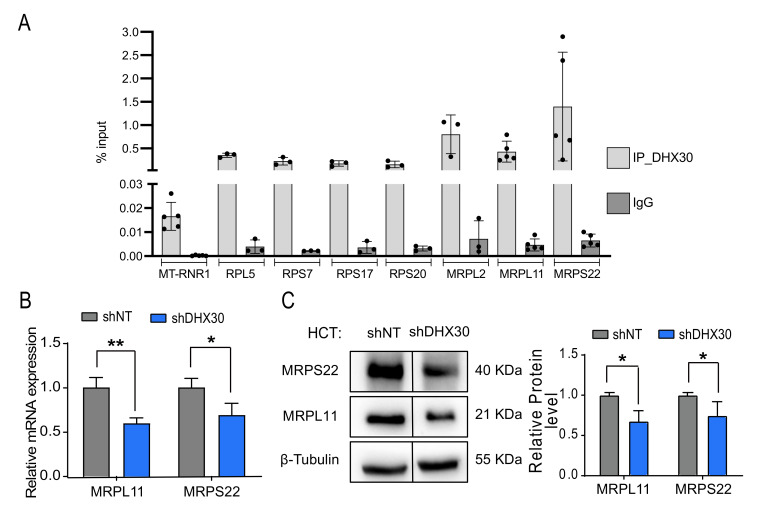
Cytoplasmic DHX30 modulates the expression of nuclear encoded mitoribosome components MRPL11 and MPRS22. (**A**) RIP experiments in parental HCT116 cells were performed to study the binding of DHX30 to the indicated RPs and MRPs transcripts. Results obtained with a primary antibody targeting DHX30 (light gray) or the IgG control (dark gray) are plotted as % of input. Data are mean and individual points (3 to 5 replicates). (**B**) Relative mRNA levels of MRPL11 and MRPS22 in HCT116_shDHX30 compared to the shNT control clone. Data are mean ± SD (n = 3); * *p* < 0.05; ** *p* < 0.01, *t*-test. (**C**) (Left) Protein levels of MRPL11 and MRPS22. β-Tubulin was used as loading control. Immunoblots represent one of three independent experiments. The vertical lines indicate that the membrane was cut to remove a Nutlin-treated sample that was not relevant to this figure. (Right) MRPL11 and MRPS22 protein quantification in HCT116_shDHX30 normalized on shNT control clone. Data are mean ± SD (n = 3); * *p* < 0.05, *t*-test. The uncropped blots are shown in supplementary materials.

The impact of DHX30 depletion on the expression and translation efficiency of nuclear-encoded mitoribosomal proteins caught our attention, as it showed the opposite trend compared to ribosomal protein transcripts (Figure 1B,C and Figure S1). We thus sought to understand if DHX30 could be directly regulating these classes of genes and exploited the data of DHX30 eCLIP assay performed in K562 cells as part of the ENCODE project [22]. Despite the different cell line, the eCLIP data indicate that DHX30 can bind 66 ribosomal (35 RPLs and 31 RPSs) and 23 mitoribosomal (16 MRPLs and 7 MRPSs) protein transcripts (Table S2) [22]. Next, we established the potential for DHX30 to bind to both ribosomal and mitoribosomal protein transcripts by RIP assays (Figure 2A), consistent with the eCLIP data mentioned above. Then, choosing MRPL11 (UL11m) that was previously evaluated in cancer cells [3] and MRPS22 (mS22) as representatives of the large and small mitoribosome subunits, we observed that the DHX30-depleted cells showed reduced expression of these two genes at both the RNA and protein levels (Figure 2B,C). Together with the TE data, these results suggest that DHX30 directly promotes the stability and/or translation of mitoribosome transcripts. The modulation of mitoribosome proteins expression might have an impact on mitochondrial translation and contribute to the functions described for DHX30 in the mitochondrial matrix [5,23]. 

### 3.3. DHX30 Protein Is Both Mitochondrial and Cytoplasmic in HCT116 Cells

**Figure 3:**
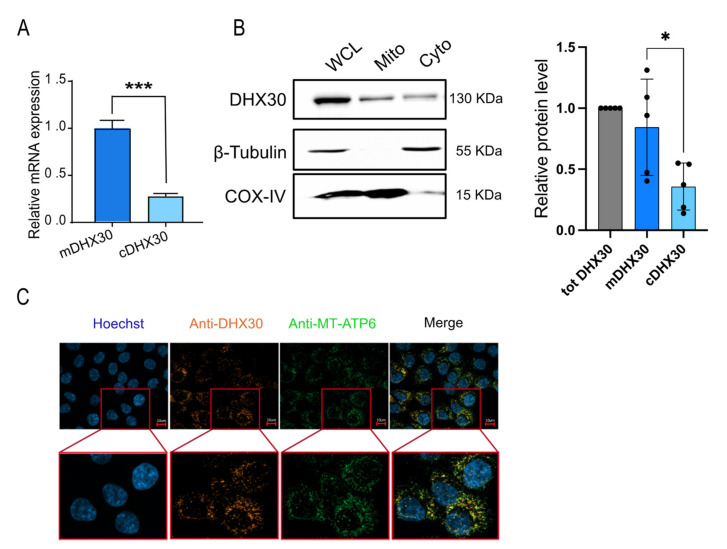
Subcellular localization of DHX30. (**A**) Relative mRNA levels of cytoplasmic DHX30 (cDHX30) and the mitochondrial variant (mDHX30, set to 1). Data are mean ± SD (n = 3); *** *p* < 0.001, *t*-test. (**B**) Relative DHX30 protein levels in whole cell lysates (WCL), mitochondrial (Mito), and cytoplasmic (Cyto) fractions. β-Tubulin and COX-IV were used as controls of cytoplasmic-mitochondrial fractionation. One of five independent experiments is shown. The graph on the right plots the relative DHX30 protein levels obtained from densitometric quantification of all the blots and relative to the reference proteins; * *p* < 0.05, *t*-test. tot DHX30: DHX30 protein in the whole cell lysate; mDHX30 and cDHX30: DHX30 protein in mitochondrial and cytoplasmic fractions, respectively. (**C**) Representative images of one of three independent immunofluorescence experiments to colocalize DHX30 (orange) with MT-ATP6 (green, used as mitochondrial marker). Staining with Hoechst (blue) was used to visualize cells’ nuclei. The uncropped blots are shown in supplementary materials.

The DHX30 gene contains two promoters, and the transcript resulting from the internal one (ENST00000457607.1) includes an alternative first exon that contains a predicted mitochondrial targeting sequence [24,25]. We checked the relative levels of the DHX30 transcripts from the two promoters and found the mitochondrial isoform to be about four times more abundant (Figure 3A). Cytoplasmic and mitochondrial fractions, as well as whole cell lysates analyzed by western blot, confirmed the higher proportion of the mitochondrial DHX30 protein (Figure 3B). Mitochondrial localization of the protein was confirmed by immunofluorescence (Figure 3C), consistent with previous results in human fibroblasts [5] and with APEX-seq data [26]. 

### 3.4. Transient DHX30 Silencing Leads to Enhanced Translation but Reduced Mitoribosome Gene Expression in HCT116 Cells

**Figure 4:**
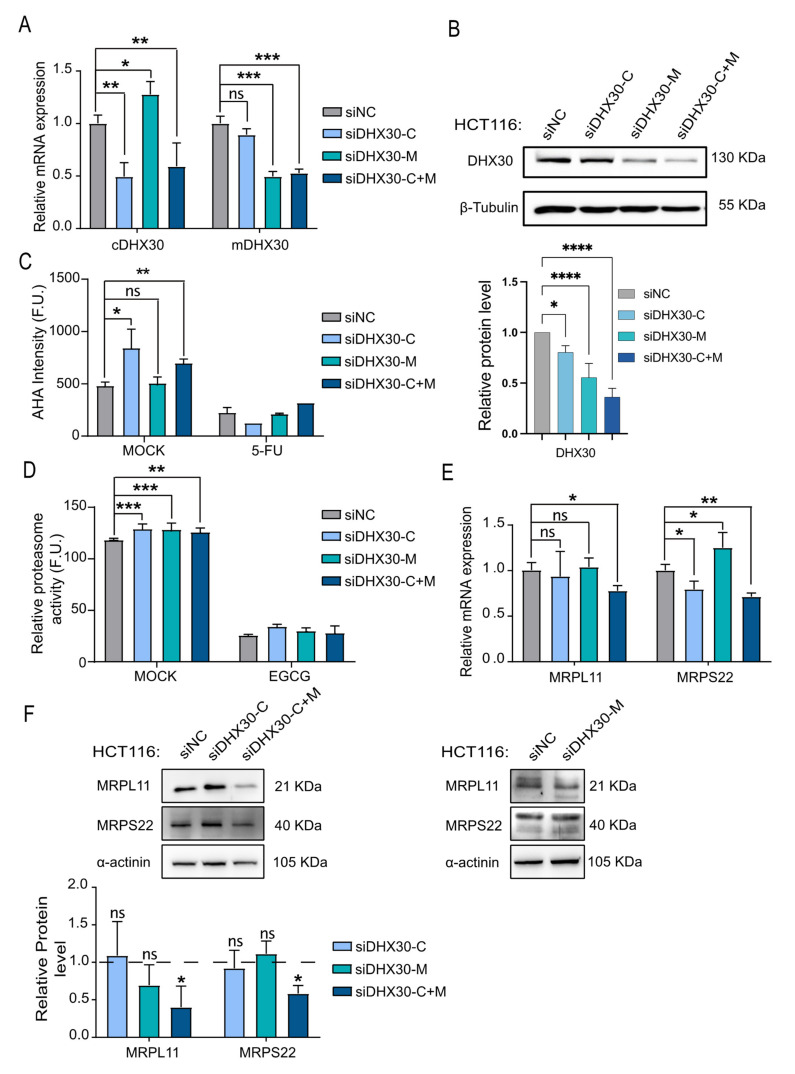
Transient DHX30 silencing leads to enhanced translation but reduced mitoribosome gene expression in HCT116 cells. (**A**) RT-qPCR to verify the silencing of DHX30 transcripts in HCT116 96 h after transient transfection with the indicated siRNAs. The relative mRNA levels of cytoplasmic (cDHX30) and mitochondrial (mDHX30) DHX30 transcripts compared to the siRNA non-targeting control (siNC) are shown. Data are mean ± SD (n = 3); ns = not significant; * *p* < 0.05; ** *p* < 0.01; *** *p* < 0.001, one-way ANOVA. (**B**) (Top) Example of a western blot comparing DHX30 protein levels in transiently silenced HCT116 cells. Tubulin was used as loading control. (Bottom) Quantification of the relative DHX30 protein levels from three (siDHX30-C) to four western blot replicates. Mean ± SD are shown; * *p* < 0.05; **** *p* < 0.0001, one-way ANOVA. (**C**) Global translation based on the fluorescence intensity of L-azidohomoalanine (AHA) incorporated in nascent proteins present in the cytoplasm. HCT116 were transiently transfected with the indicated siRNAs and tested after 96 h. 5-Fluorouracil treatment was used as control treatment leading to translation inhibition. Data are mean ± SD (n = 3); ns = not significant; * *p* < 0.05; ** *p* < 0.01, one-way ANOVA. (**D**) Relative proteasome activity expressed as fluorescence units in transiently silenced HCT116 cells (legend as in panel **C**) in the mock condition or after treatment with epigallocatechin gallate (EGCG) used as a 20S proteasome inhibitor. Data are plotted as mean ± SD (n = 3); ns = not significant; ** *p* < 0.01; *** *p* < 0.001, one-way ANOVA. (**E**) mRNA levels of MRPL11 and MRPS22 in HCT116 silenced transiently with the indicated siRNAs. Mean ± SD (n = 3); * *p* < 0.05, ** *p* < 0.01, one-way ANOVA. (**F**) (Top left) MRPL11 and MRPS22 protein levels in HCT116 silenced for cytoplasmic DHX30 (siDHX30-C) or for both cytoplasmic and mitochondrial variants (siDHX30-C+M). (Bottom left) MRPL11 and MRPS22 protein levels in HCT116 silenced for mitochondrial DHX30 (siDHX30-M). Immunoblot represents one of three independent experiments. (Right) Relative protein quantification normalized on α-actinin and compared to siNC control (set to 1). Data are mean ± SD (n = 3); ns = not significant; * *p* < 0.05, *t*-test. The uncropped blots are shown in supplementary materials.

Since stable DHX30-silencing was achieved using three different shRNAs [7] that targeted exons common to both cytoplasmic and mitochondrial transcripts (from here on labeled cDHX30 and mDHX30) (see methods for details), we next decided to confirm and extend our analysis using isoform-specific transient silencing. We performed transient silencing experiments using one siRNA specific for cDHX30 (labeled siDHX30-C), one siRNA specific for mDHX30 (labeled siDHX30-M), and a third siRNA targeting an exon present in the transcripts produced from both promoters (labeled siDHX30-C+M). The activity and specificity of the siRNAs were assessed by RT-qPCR 96 h post-silencing (Figure 4A) and by western blot (Figure 4B).

Overall, transient silencing was slightly less effective based on the comparison of residual DHX30 protein expression with the stably depleted clones (Figure S1H). Nevertheless, the siDHX30-C+M reduced the expression down to about 35% (Figure 4B), and, considering the relative proportion of cytoplasmic and mitochondrial DHX30 protein in HCT116 cells (Figure 3B), it can be estimated that both siDHX30-C and siDHX30-M reduce DHX30 protein in their specific compartment to about 50%. The transient silencing of just cDHX30 or both cDHX30 and mDHX30, but not of only mDHX30, led to an increase in global translation (Figure 4C) that, however, was not associated with an increase in the activity mTOR pathway (Figure S2A). Hence, these results not only confirmed the observations obtained with the stable shRNA clones but indicated that it is cDHX30 that can modulate global translation. A slight increase in proteasome activity was observed for all three DHX30 siRNAs compared to the non-targeting control (Figure 4D). The simultaneous transient silencing of both cDHX30 and mDHX30 resulted in a reduction of the expression of MRPL11 and MRPS22 (Figure 4E,F) similar to what we observed with the stably DHX30 depleted cells (Figure 2B,C). Conversely, the silencing of only cDHX30 and, particularly, of only mDHX30 were ineffective, considering the results of both RNA and protein levels for MRPL11 and MRPS22 (Figure 4F).

### 3.5. DHX30 Depletion Impacts Mitochondrial Gene Expression and Function

**Figure 5:**
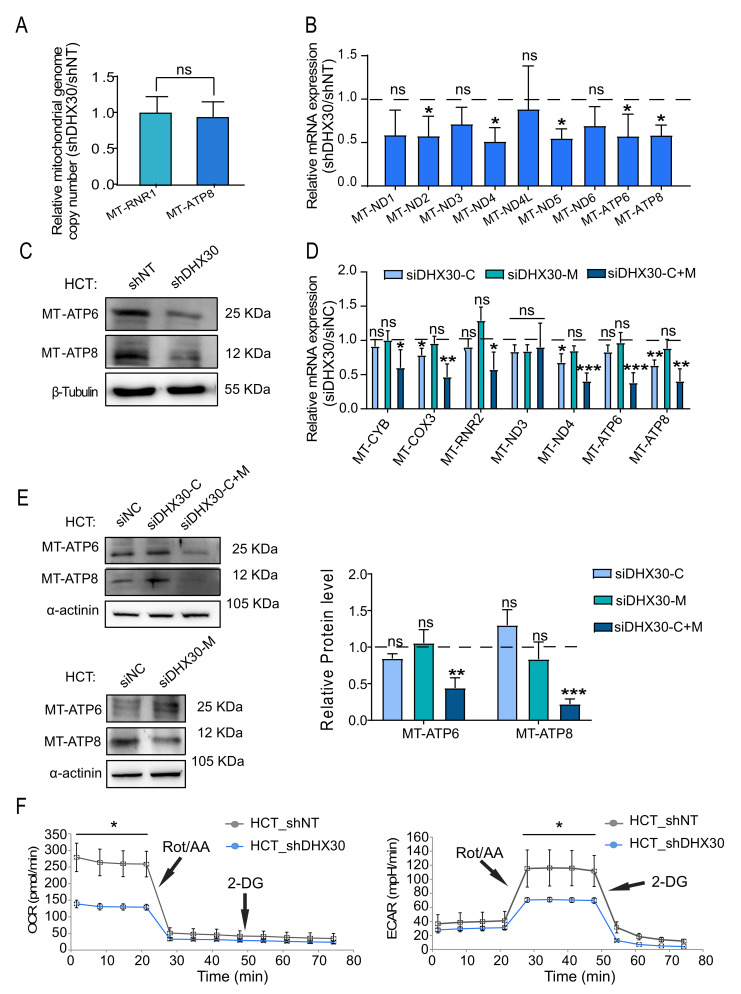
Depletion of DHX30 reduces the expression and function of mitochondrially encoded OXPHOS components. (**A**) Relative mitochondrial genome copy number in HCT116_shDHX30 and _shNT measured by droplet digital PCR. MT-RNR1 and MT-ATP8 were amplified along with the nuclear diploid marker gene CTDSP1. Bars plot mean ± SD (n = 3), *t*-test; ns = not significant. (**B**) Relative mRNA levels of mitochondrially encoded OXPHOS components in HCT116_shDHX30 compared to HCT116_shNT (dashed line, set to 1). Data are mean ± SD (n = 3); ns = not significant; * *p* < 0.05, *t*-test. (**C**) Protein levels of MT-ATP6 and MT-ATP8 in the mitochondrial lysates of HCT116_shDHX30 and the shNT control clone. β-Tubulin was used as loading control. Immunoblots represent one of two independent qualitative comparisons. (**D**) Relative mRNA levels of the indicated mitochondrially encoded OXPHOS components in HCT116 transiently silenced for DHX30 expression (siDHX30-C, siDHX30-M, and siDHX30-C+M) compared to siNC (dashed line, set to 1). Data are mean ± SD (n = 3); ns = not significant; * *p* < 0.05; ** *p* < 0.01; *** *p* < 0.001, *t*-test. (**E**) (Left) Protein levels of MT-ATP6 and MT-ATP8 in HCT116 transiently silenced as in (D). α-actinin was used as loading control. Immunoblots represent one of three independent experiments. (Right) Relative MT-ATP6 and MT-ATP8 protein quantification normalized on α-actinin and compared to siNC control. Data are mean ± SD (n = 3); ns = not significant; ** *p* < 0.01; *** *p* < 0.001, *t*-test. (**F**) (Left) Measurement of the oxygen consumption rate (OCR) to evaluate mitochondrial respiration by the Seahorse XF analyzer. Points before the Rotenone/Antimycin-A (Rot/AA) treatment correspond to the basal mitochondrial respiration. (Right) Extracellular acidification rate (ECAR) measurement was used to measure glycolysis. Points before and after the Rotenone/Antimycin-A (Rot/AA) treatment correspond respectively to basal and compensatory glycolysis in response to the block of mitochondrial respiration. 2-Deoxyglucose (2-DG) is then used to block glycolysis. For both panels, one of three independent replicates is presented. Data are mean ± SD (n = 3 wells in the Seahorse cartridge); * *p* < 0.05, *t*-test. The uncropped blots are shown in supplementary materials.

We next focused on the expression of mitochondrially encoded genes. First, we established that DHX30 depletion did not have an impact on the number of mitochondrial genomes in HCT116 cells by digital PCR (Figure 5A). Instead, the expression of several mitochondrially encoded transcripts was significantly reduced (Figure 5B). The steady-state expressions of two mitochondrially encoded proteins, MT-ATP6 and MT-ATP8, were investigated and shown to be reduced in HCT116_shDHX30 cells (Figure 5C and Figure S2B). We pursued the same analysis after transient silencing, confirming the lower expression of mitochondrially encoded genes at the RNA (seven targets tested) and protein (for MT-ATP6 and -ATP8) levels but only when both cytoplasmic and mitochondrial DHX30 were silenced (siDHX30-C+M) (Figure 5D,E).

Finally, we evaluated the markers of carbon metabolism and mitochondrial respiration capacity by an Agilent SeahorseXF real-time analyzer. HCT116_shDHX30 cells had a significantly lower basal oxygen consumption rate compared to the control cell line. Notably, the DHX30-depleted cells did not show compensatory glycolysis in basal culture condition, not even when we chemically abolished mitochondrial respiration. We indeed observed a lower compensatory glycolysis with respect to the HCT116_shNT control (Figure 5F). These results suggest that HCT116_shDHX30 cells do not show a typical Warburg effect [27] and are expected to produce less energy due to reduced mitochondrial respiration. 

Collectively, the depletion of DHX30 seems to generate an imbalance in cell homeostasis, with a higher demand of chemical energy from ribosome biogenesis and cytoplasmic translation but lower mitochondrial activity.

### 3.6. DHX30 Depletion Impaired Cell Proliferation Rate and Increased Apoptosis Proneness

**Figure 6:**
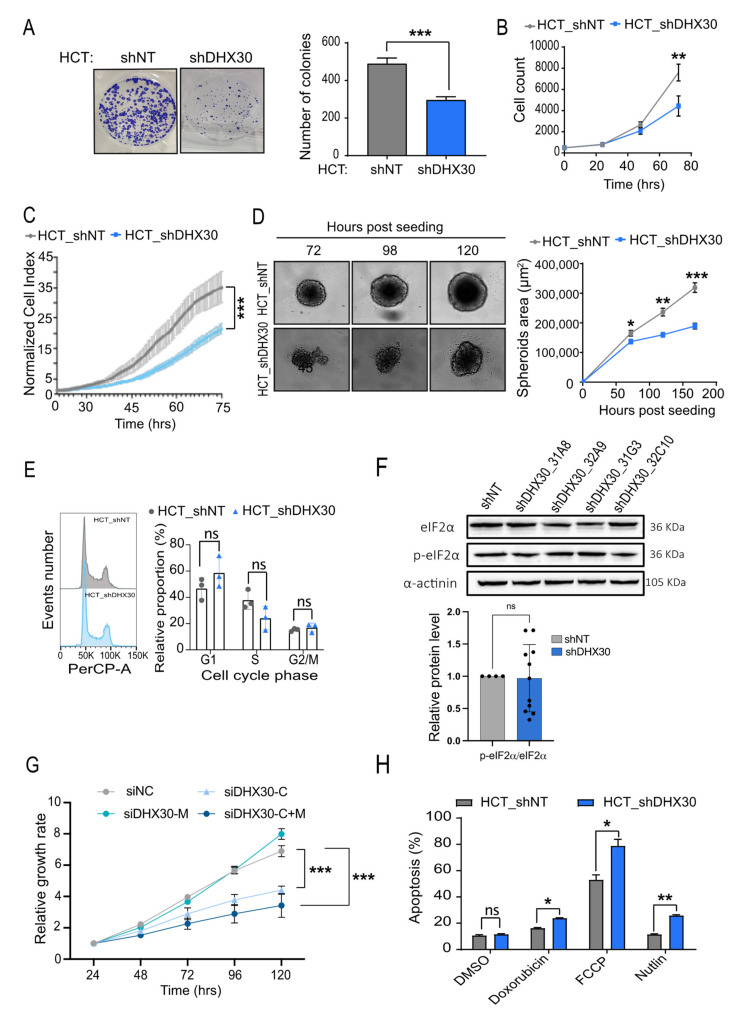
Depletion of DHX30 reduces proliferation and survival in basal and treatment conditions. (**A**) (Left) Representative images of colony formation assays. (Right) Colony quantification by ImageJ software. Data are mean ± SD (n = 3) *** *p* < 0.001, *t*-test. (**B**) Relative cell proliferation measured by high-content microscopy in digital phase contrast. Data are mean ± SD (n = 3; ** *p* < 0.01, two-way ANOVA. (**C**) Estimate of relative proliferation by an impedance-based Real-Time Cell Analyzer. Data are mean ± SD (n = 3); *** *p* < 0.001, two-way ANOVA. (**D**) Spheroid formation and growth assay. (Left) A representative image at the indicated time points is shown in the left panel. (Right) Spheroid area measured by ImageJ software. Data are mean ± SD (n = 3); * *p* < 0.05; ** *p* < 0.01; *** *p* < 0.001, two-way ANOVA. (**E**) (Left) Representative image of HCT116_shNT and shDHX30 cell cycle profiles. (Right) Quantification of cell cycle profile in HCT116 DHX30 depleted cells compared to shNT control. Data are mean ± SD (n = 3); ns = not significant, *t*-test. (**F**) (Top) Representative immunodetection image of eIF2α and phosho-eIF2α in the indicated HCT116 cell clones stably depleted for DHX30 expression or the control shNT. α-actinin was used as loading control. (Bottom) Quantification of relative phosho-eIF2α protein levels. Mean + SD and individual values (n = 11) are shown, *t*-test; ns = not significant. (**G**) Relative proliferation measured as in (B) but starting 24 h after transient silencing DHX30 with the indicated siRNAs *** *p* < 0.001, two-way ANOVA. (**H**) Relative expression of the annexin-V apoptosis markers in cells treated for 48 h with the indicated drugs or DMSO control. Data are mean ± SD (n = 3); ns = not significant; * *p* < 0.05; ** *p* < 0.01, *t*-test. The uncropped blots are shown in supplementary materials.

Next, we characterized the phenotypic impact of DHX30 depletion. Consistent with the lower mitochondrial oxygen consumption rate, HCT116_shDHX30 cells showed a significantly lower proliferation rate compared to the control cell clone. This was observed by colony formation assay and confirmed through time-course proliferation by cell count via high-content microscopy or using a Real-Time Cell Analyzer (Figure 6A–C and Figure S2C). Interestingly, we observed a reduction in proliferation after DHX30 silencing also in a spheroid assay (Figure 6D). The lower growth rate in DHX30 depleted cells could not be attributed to an overt arrest in a particular phase of the cell cycle, although a trend for a higher proportion of G1 cells was apparent (Figure 6E). Neither could it be associated with an increase in the phosphorylation of eIF2alpha (Figure 6F) nor to the activation of ER-stress response, based on the expression of CHOP, DDIT4, TRIB3, and the XBP1 splicing pattern (Figure S2D).

A reduction in proliferation was also confirmed by cell counting upon the transient silencing of cDHX30 and, particularly, of combined cDHX30 and mDHX30. Interestingly, the silencing of only mitochondrial DHX30 did not have an impact on proliferation at the time points tested (Figure 6G).

Furthermore, DHX30 depletion was associated with increased apoptosis after 48 h of Nutlin treatment, as expected by our previous results [6], but also when cells were treated with the topoisomerase inhibitor doxorubicin and with FCCP, an agent that causes mitochondrial membrane depolarization (Figure 6H).

### 3.7. DHX30 Depletion Enhances Global Translation but Reduces Proliferations also in U2OS and MCF7 Cells

The analysis of DHX30 stable or transient silencing (siDHX30-C or siDHX30C+M) was extended to U2OS (Figure S3A–D), osteosarcoma-derived cells that express relatively high levels of DHX30 [7]. The stable depletion of both cDHX30 and mDHX30 was associated with a significant reduction of MRPL11 (RNA and protein) and of MRPS22 mRNA expression (Figure S3E,G), while transient depletion by siRNAs did not significantly affect the expression of the two mitoribosome transcripts (Figure S3F), although MRPL11 protein levels were reduced by siDHX30-C+M (Figure S3H).

The expression of mitochondrially-encoded genes was then assessed in U2OS_shDHX30 cells. All the seven tested transcripts, except for MT-ND6, were down-modulated compared to the U2OS_shNT control (Figure S3I, left panel). Instead, none of the seven transcripts were downmodulated by transient DHX30 silencing, regardless of the siRNA used (Figure S3I, right panel). In fact, three of them were slightly upregulated. This latter result may be related to the observation of lower efficacy of DHX30 depletion by the siRNAs and with the lack of an impact on MRPL11 or MRPS22 expression. MT-ATP6 was also examined by western blot, and its amount was reduced in U2OS_shDHX30 cells (Figure S3J) but not in U2OS cells that were transiently silenced for DHX30 (Figure S3K). 

The proliferation of U2OS_shDHX30 cells was strongly reduced compared to the U2OS_shNT control (Figure S4A). Unexpectedly, transient silencing did not affect the proliferation of U2OS cells, although this was consistent with the results observed on DHX30 targets gene expression (Figure S4B). However, the depletion of cDHX30 was associated with higher global translation (Figure S4C), similar to what we observed in HCT116. We cannot exclude that the strong impact of the shRNA on proliferation is influenced by off-target effects, nor that the lack of an effect for the siRNAs is due to the magnitude and duration of the transient silencing in U2OS cells.

Transient DHX30 silencing was also performed in the breast cancer-derived MCF7 cells and shown to be effective and isoform specific (Figure S4D,E). As for HCT116 cells, the negative impact on proliferation was related to the silencing of cytoplasmic DHX30 alone or to the combined DHX30 silencing (by siDHX30 C+M) (Figure S4F), which was also associated with an increase in global translation (Figure S4G). However, the silencing of mitochondrial DHX30 was also associated with an increase in global translation, different from what was observed in HCT116 cells.

### 3.8. A Candidate Mitoribosomal DHX30 Signature

Our results suggest that DHX30 exerts a constitutive function that improves cellular fitness by balancing energy metabolism and global translation potential. Furthermore, our previous study identified DHX30 as a negative modulator of the translation of specific mRNAs, thus controlling p53-dependent apoptosis [7,28]. Both of these functions suggest that DHX30 could be a modifier of cancer cell properties, potentially impacting clinical variables.

Although total RNA-seq data are not a good proxy for investigating translation controls [29], in this study, we showed that DHX30 depletion impacts the steady-state levels of nuclear-encoded mitoribosomal transcripts. We next integrated our differential TE and GSEA enrichment data with the DHX30 targets found by our RIP and DHX30 eCLIP data in ENCODE [22]. By this intersection, we compiled a list of 14 mitoribosomal protein (MRP) transcripts that we consider high-confidence candidate DHX30 direct targets (Figure S5A,B). We eventually used this candidate signature for the preliminary assessment of its potential prognostic value through the GEPIA web resource [20,21] (Figure S5C,D and S6), whose results warrant further work to explore its potential association with prognosis in several cancer types. Finally, a pan-tissue view revealed a general positive correlation between the expression of ribosome and mitoribosome protein transcripts in normal samples that is reduced in cancer (Figure S7). These preliminary analyses suggest that a functional signature could be developed to predict aggressiveness in cancer types where DHX30 appears to stimulate mitoribosomal proteins expression.

## 4. Discussion

Ribosome biogenesis and translation impose a high metabolic demand on the cell [30,31]. Hence, a coordination between translational control and metabolic output ultimately involving mitochondrial respiratory functions is expected to contribute to cell homeostasis and fitness [32,33]. However, relatively few proteins and pathways have been established to exert a direct role in balancing cytoplasmic translation initiation with mitochondrial metabolism [34,35]. eIF6 represents a significant example, as it has been clearly shown that it negatively controls 80S monosome assembly, a necessary step for translation initiation, while at the same time playing a critical positive role in mitochondrial functions [36,37,38].

We propose that DHX30 can also exert an important housekeeping role in coordinating ribosome biogenesis, translation, and mitochondrial respiration. DHX30 depletion in HCT116, stable or transient, leads to a modest but significant increase in ribosome biogenesis as well as in global translation; on the contrary, mitoribosome protein transcripts, particularly of the large subunits, exhibit reduced translation efficiency. Furthermore, DHX30 can also exert a more direct role on mitochondria, as the protein can directly localize to the organelle thanks to an alternative first exon that features a localization signal. The steady state expression of several mitochondrially encoded genes was reduced following the depletion of both DHX30 transcripts. A previous study provided clear evidence that DHX30 together with DDX28, FASTKD2, and FASTKD5 can promote the assembly of 55S mitoribosome and translation [5]. Consistent with the relevance of a mitochondrial and translation function, the DHX30 transcript has been shown to be located and locally translated at the ER-Outer-Mitochondrial membrane interface by APEX-seq [26]. In fact, none of the DHX30 closer homologs showed strong evidence of such localized translation or other evidence of mitochondrial localization.

A previous report also showed evidence for DHX30 interaction with mitochondrial transcripts in human fibroblasts by RIP-seq [5]. Our data instead point to a direct interaction with mitoribosome transcripts and their positive modulation as another means by which DHX30 can indirectly affect mitochondrial translation. We validated by RIP the binding of DHX30 to MRPL2, MRPL11, and MRPS22. Leveraging public data from eCLIP experiments in ENCODE and our polysomal profiling, RNA-seq, and GSEA analysis, we propose that DHX30 could directly interact with fourteen mitoribosome protein transcripts. An even larger set of cytosolic ribosomal protein transcripts are nominated as direct DHX30 targets by eCLIP [22] and are showing changes in translation efficiency upon DHX30 depletion in HCT116 cells. 

We observed that these two groupings of transcripts markedly differed for their basal translation efficiency, which was low for cytoplasmic ribosomal protein transcripts. Those mRNAs are known to contain particularly structured 5′-UTR and to be strongly regulated at the level of translation initiation by mTOR and MYC-regulated pathways [39,40,41]. The mechanism by which DHX30 can control ribosomal protein (RP) transcripts remains to be established and could also be dependent on the regulation of transcription or mRNA stability. In a recent study, we discovered an RNA sequence motif in 3′UTRs, labeled CGPD, that is bound by DHX30 and can mediate higher translation levels [7]. While we cannot completely exclude that the CGPD motif could be implicated, only a subset of RP transcripts harbors instances of it. Our de novo search for over-represented motifs did not retrieve another prominent candidate *cis*-element. An even lower number of mitoribosome protein transcripts (MRPs) were found to harbor instances of the CGPD-motif. This was expected, as DHX30 is inferred to have an opposite role on RP and MRP transcripts based on the direction of changes in their TE observed in HCT116 shDHX30 cells. For the MRP gene group, a de novo motif over-representation search did not identify a strongly enriched *cis*-element. 

The magnitude of fold-change and translation efficiency changes for both ribosomal and mitoribosomal transcripts in response to DHX30 silencing is modest in absolute value. This could be in part due to a limitation of the experimental approach. As reported in our earlier study, a complete knock-out of DHX30 does not seem to be attainable in HCT116 cells. The shRNA clones and, particularly, the transiently silenced cells by siRNAs still retain partial DHX30 protein expression. In addition, the fact that one siRNA for isoform-specific silencing was used represents a limitation of the study, given the possibility of off-target effects. However, the overall results were consistent among the stable and transient combined depletion of both cytoplasmic and mitochondrial DHX30 isoforms. We also noted possible differences between HCT116 and MCF7 cells for the effect of mitochondrial DHX30 silencing that will need to be further explored. Furthermore, it is important to emphasize that changes in the expression or even polysomal association of ribosomal protein transcripts do not necessarily predict translation rate changes. However, several endpoints consistently suggested that HCT116_shDHX30 cells exhibited higher ribosome number and increased global translation. When treated with an MDM2 inhibitor inducing a strong p53-dependent transcriptional response, those cells were also shown to markedly alter their translatome [7,28]. Our results pointed to a direct role of DHX30 on translation specificity. However, we cannot exclude that the global effect on ribosome biogenesis we observed represents a constitutive, housekeeping function for DHX30. This function would also contribute to the observed changes in translation specificity, under the notion that a change in global translation potential or in the modulation of translation initiation is not expected to equally impact every available transcript in a cell [33,42].

The depletion of DHX30 reduced cell proliferation in various assays. This effect was also visible by the transient silencing of just the cytoplasmic transcript, although it was more evident when both cytoplasmic and mitochondrial isoforms were silenced. Instead, the silencing of only the mitochondrial DHX30 had no clear impact on proliferation nor on global translation at the time points followed in the experiments. Although we cannot exclude that the lower proliferation results from a checkpoint activation, the cells did not show evidence for overt cell cycle arrest. This is not entirely unexpected due to the residual levels of DHX30 expression in the cell models we used, as noted above, and also for the possible selective pressure, particularly in stably depleted clones, for compensatory effects given the central nature of the processes involved.

The expression of several mitoribosome components has been evaluated as potential biomarkers associated with cancer clinical variables [43]. As several other RBPs, mitoribosomal proteins have been proposed to have additional, moonlighting functions unrelated to mitoribosome biogenesis—particularly, in the modulation of apoptosis. For example, MRPS29, also known as DAP3, was reported to influence the extrinsic apoptosis pathway [44,45], while MRPL41 was proposed to modulate p53-dependent intrinsic apoptosis [46]. It is, however, unlikely that these potential pro-apoptotic functions can contribute to the proliferation defects seen after DHX30 depletion, given that their expression is not upregulated.

Hence, we reasoned that changes in DHX30 levels to an extent that would not lead to overt stress responses could provide opportunities for increased fitness due to the coordination between mitochondrial function and translation potential, which could be a balancing function between energy supply and demand. If any, the effects would be expected to reflect tissue-specific differences in metabolism. Integrating eCLIP data with our polysomal profiling, RNA-seq, RIP results, and GSEA functional enrichment data, we compiled a list of fourteen mitoribosomal transcripts that we consider high-confidence candidate direct DHX30 targets. Preliminary analyses suggest that the combined 15-gene list could be relevant to prognosis in several cancer types, suggesting an avenue of investigation for future research.

## 5. Conclusions

Our study identifies a role for DHX30 in the coordination between global cytoplasmic translation and mitochondrial functions. This role contributes to the oncogenic potential of cancer cells and appears to correlate with tumor aggressiveness and clinical outcomes. Furthermore, during stress responses activating p53, DHX30 can reduce the apoptotic commitment of cancer cells by acting on specific pro-apoptotic transcripts, thus providing a potential actionable target for therapeutic purposes [7].

## Data Availability

The RNA-seq datasets are available in GEO with ID GSE95024 and GSE154065.

## Supplementary Materials

The following are available online at https://www.mdpi.com/article/10.3390/cancers13174412/s1, Figure S1: Relative expression of ribosomal and mitoribosomal protein transcripts, efficacy of DHX30 shRNAs, status of mTOR and MYC pathways, association of DHX30 protein with ribosomes and polysomes, and analysis of global translation. Related to Figure 1. Figure S2: Impact of transient DHX30 silencing in HCT116 on the activation of the MTOR pathway, of stable DHX30 silencing on the expression of the MT-ATP6, on cell proliferation, and on the expression of ER-stress response genes. Related to Figure 4, Figure 5 and Figure 6. Figure S3: Impact of transient or stable DHX30 silencing in U2OS cells on mitoribosome protein transcripts or on mitochondrially encoded transcripts. Figure S4: Impact of stable or transient, isoform specific DHX30 silencing on cell proliferation and global translation in U2OS and MCF7 cells. Figure S5: DHX30 expression positively correlates with the expression of mitoribosomal protein transcripts, and a derived 15-gene signature could have prognostic significance. Figure S6: Expression of DHX30 in TCGA tumor types and matched healthy tissues. Figure S7: Expression correlation of cytoplasmic and mitochondrial ribosome component genes in TCGA and GTEX samples. Table S1: RNA-seq Fold Change data and results of GSEA analysis for the comparison between HCT_shDHX30 and HCT_shNT. Table S2: Ribosomal and mitoribosomal protein mRNAs bound by DHX30 in the ENCODE HepG2 eCLIP dataset. Table S3: siRNAs, FISH probe, and primer sequences. Table S4: List of primary and secondary antibodies.
