# Supplementary material for: DHX30 Coordinates Cytoplasmic Translation and Mitochondrial Function Contributing to Cancer Cell Survival
Source: Cancers (Basel). 2021 Aug 31;13(17):4412. doi: 10.3390/cancers13174412 (PMC8430983; doi:10.3390/cancers13174412)

Figure 2C

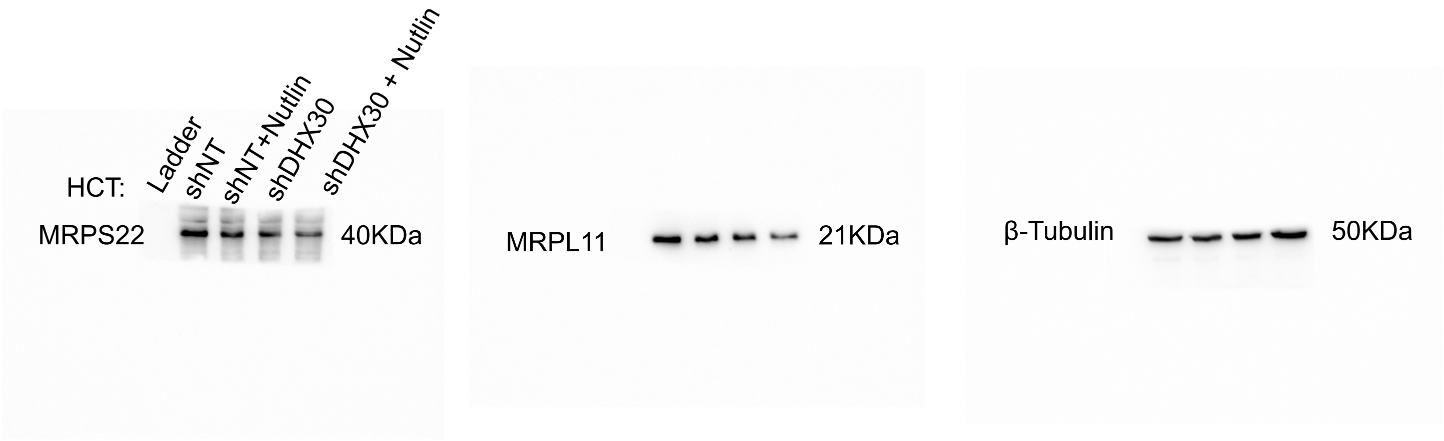

Figure 3B

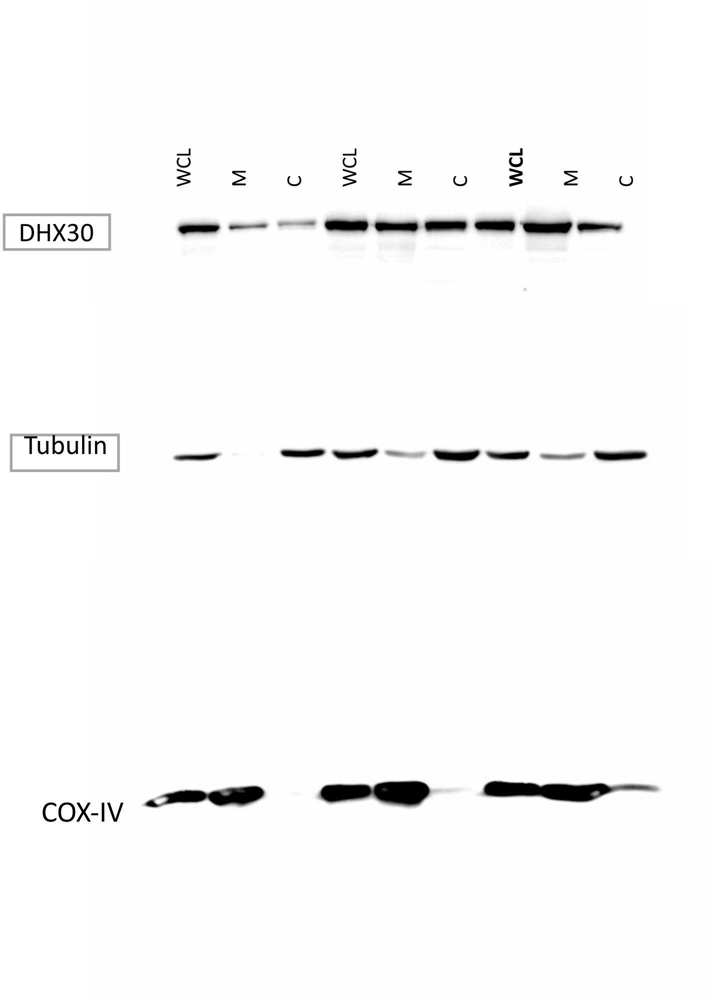

Figure 4B

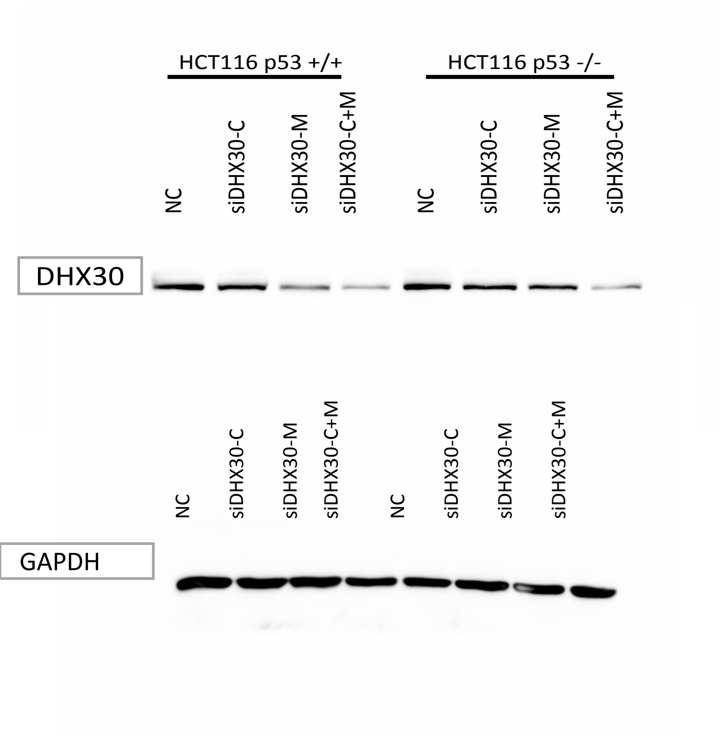

Figure 4F-Up

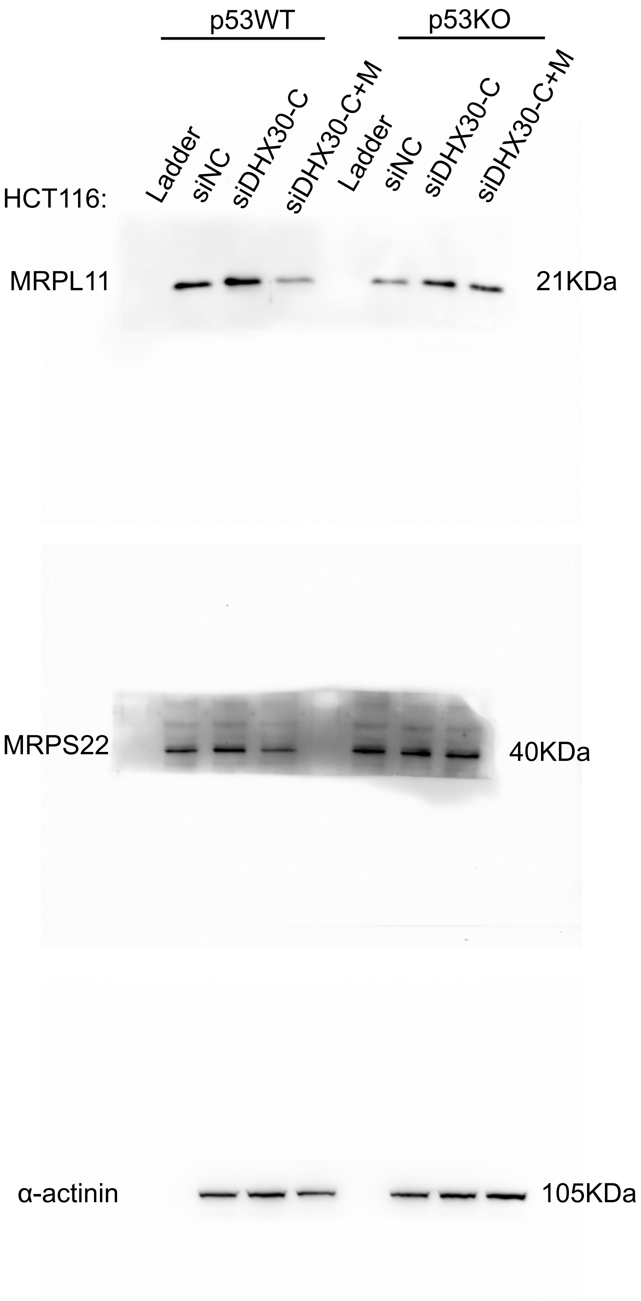

Figure 4F-Down

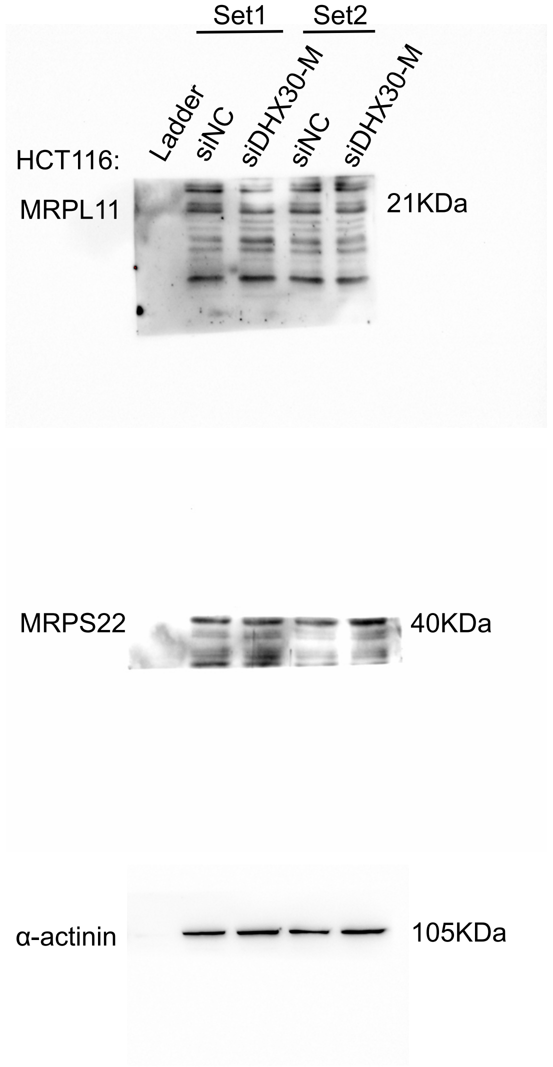

Figure 5C

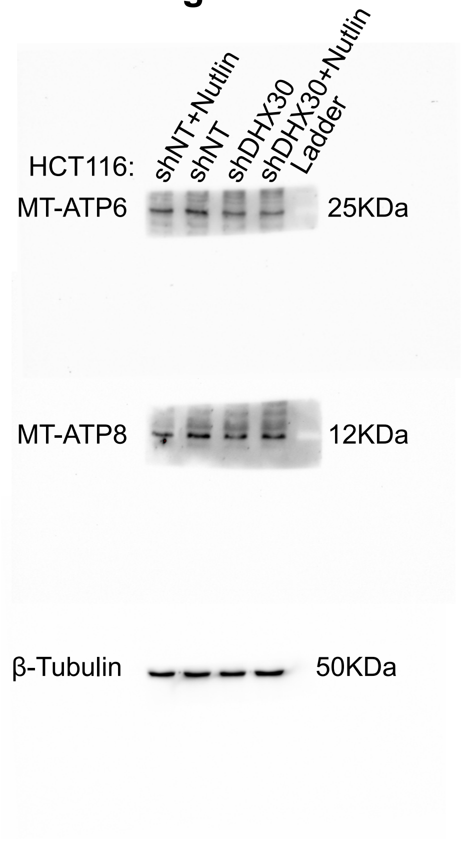

Figure 5E-Up

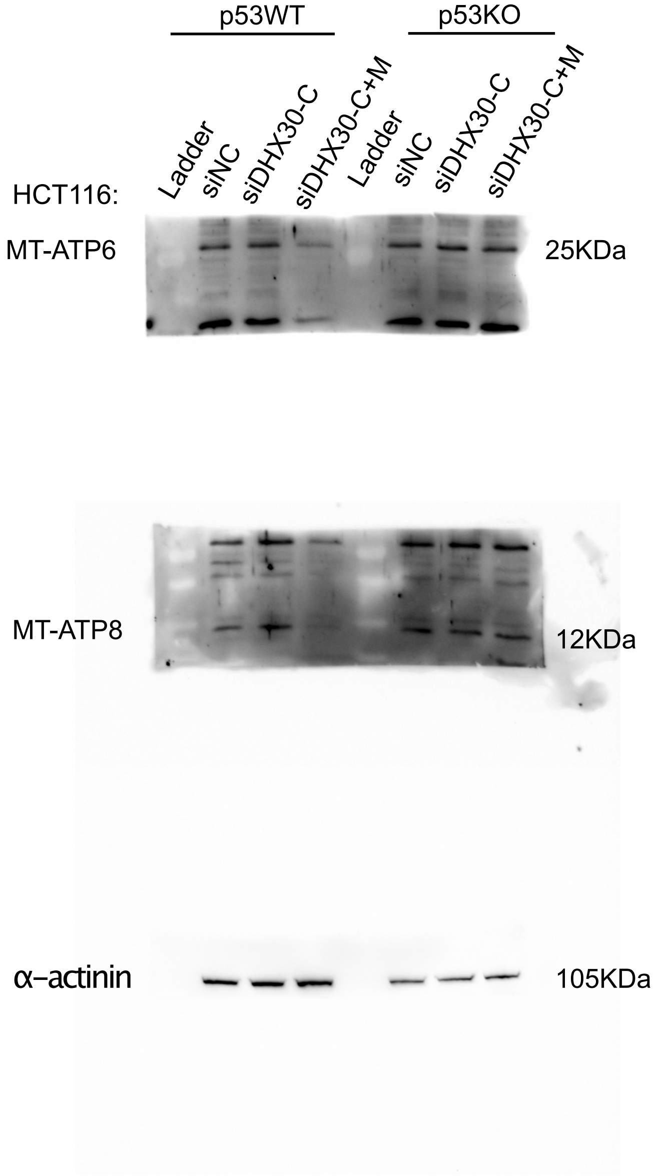

Figure 5E-Down

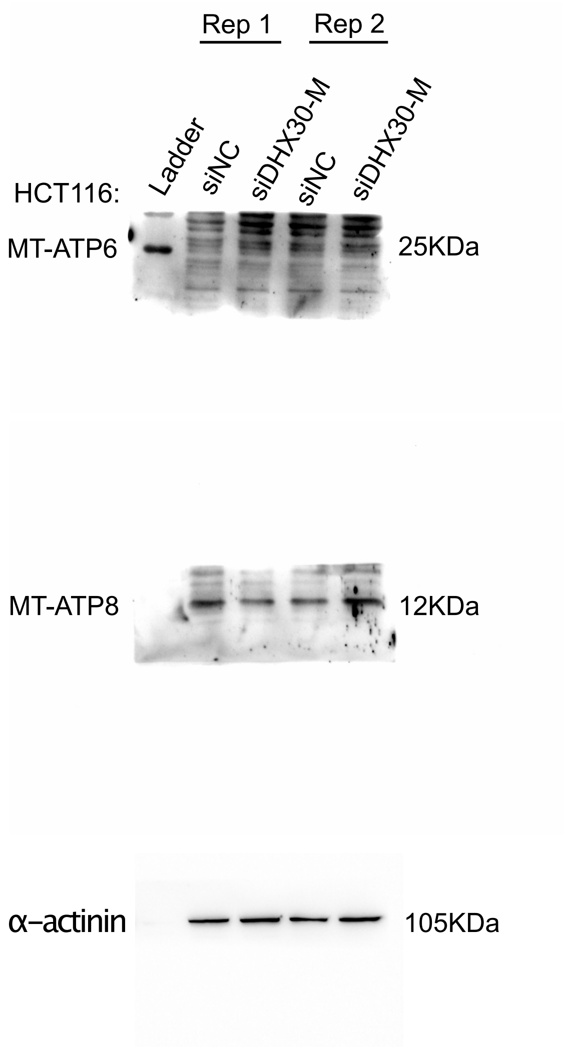

Figure 6F

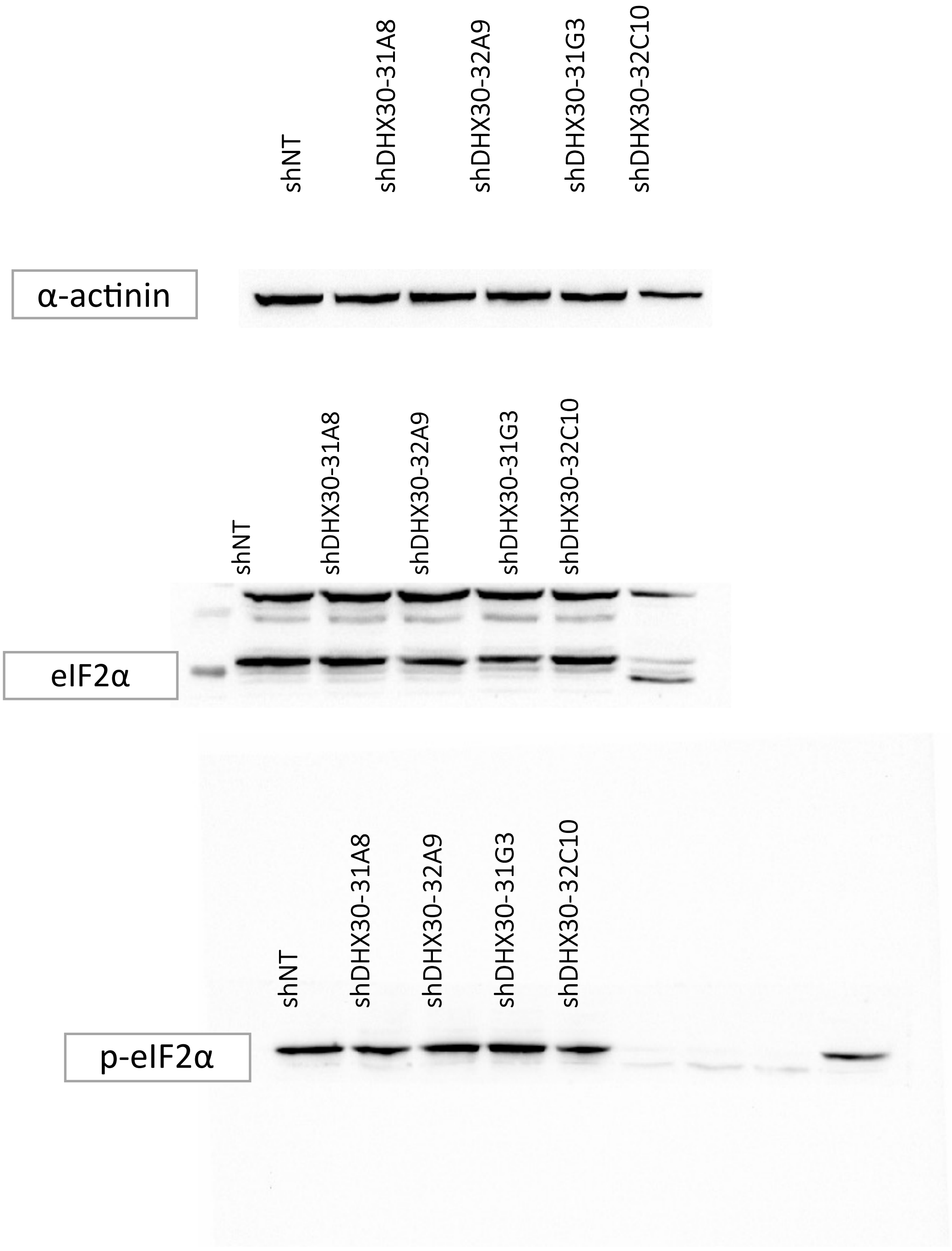
shNT
shDHX30-31A8
shDHX30-32A9
shDHX30-31G3
shDHX30-32C10
α-actinin
shNT
shDHX30-31A8
shDHX30-32A9
shDHX30-31G3
shDHX30-32C10
eIF2α
shNT
shDHX30-31A8
shDHX30-32A9
shDHX30-31G3
shDHX30-32C10
p-eIF2α

Figure S1C

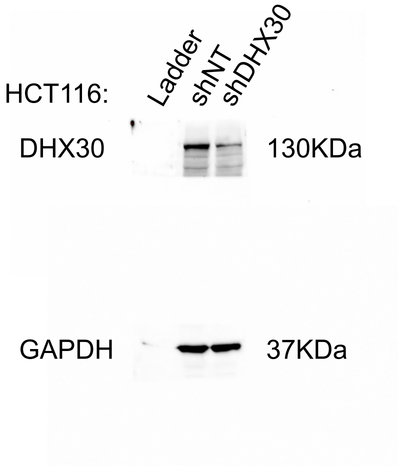

Figure S1E

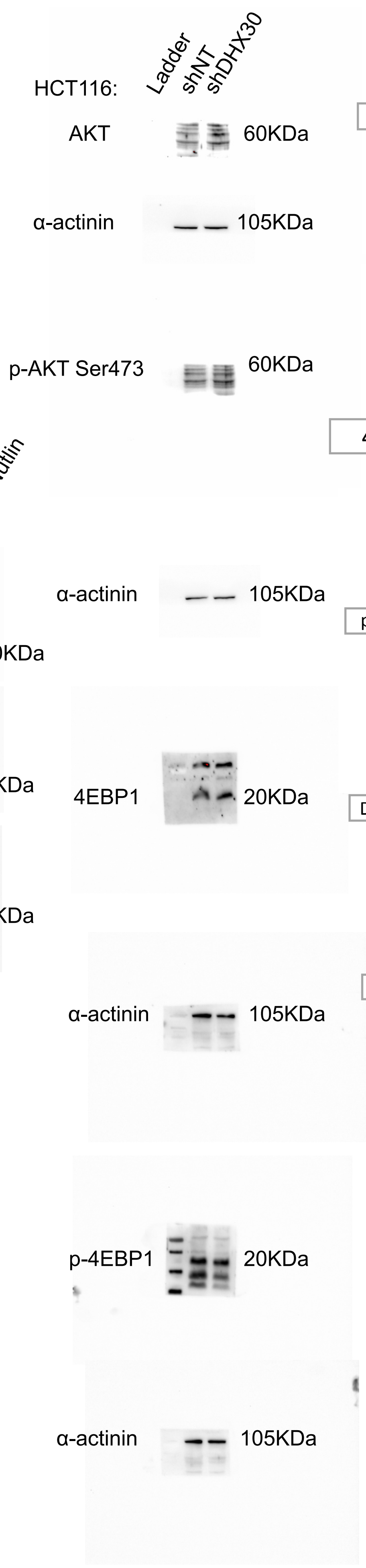

Figure S1F

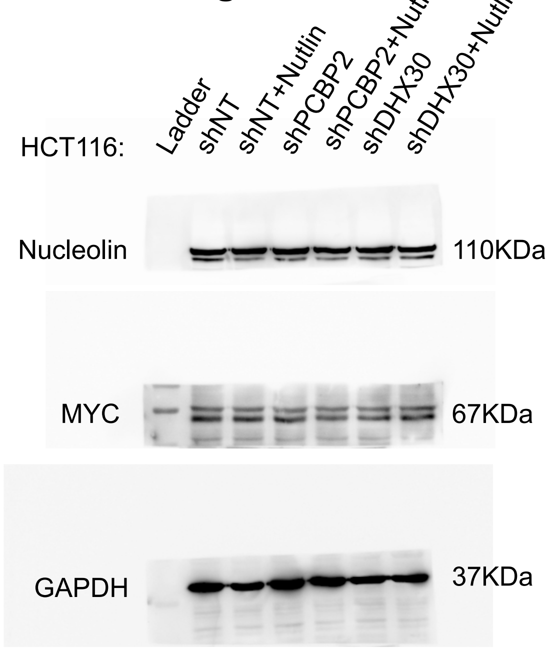

Figure S1H

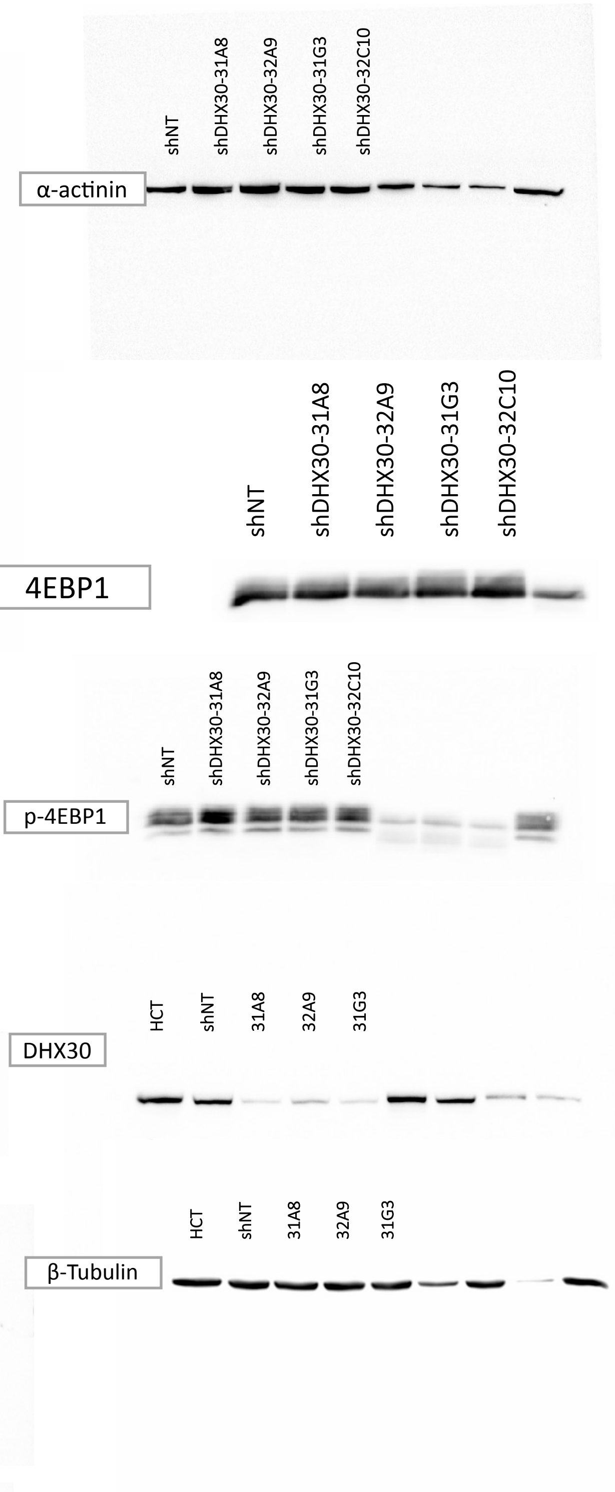

Figure S2A

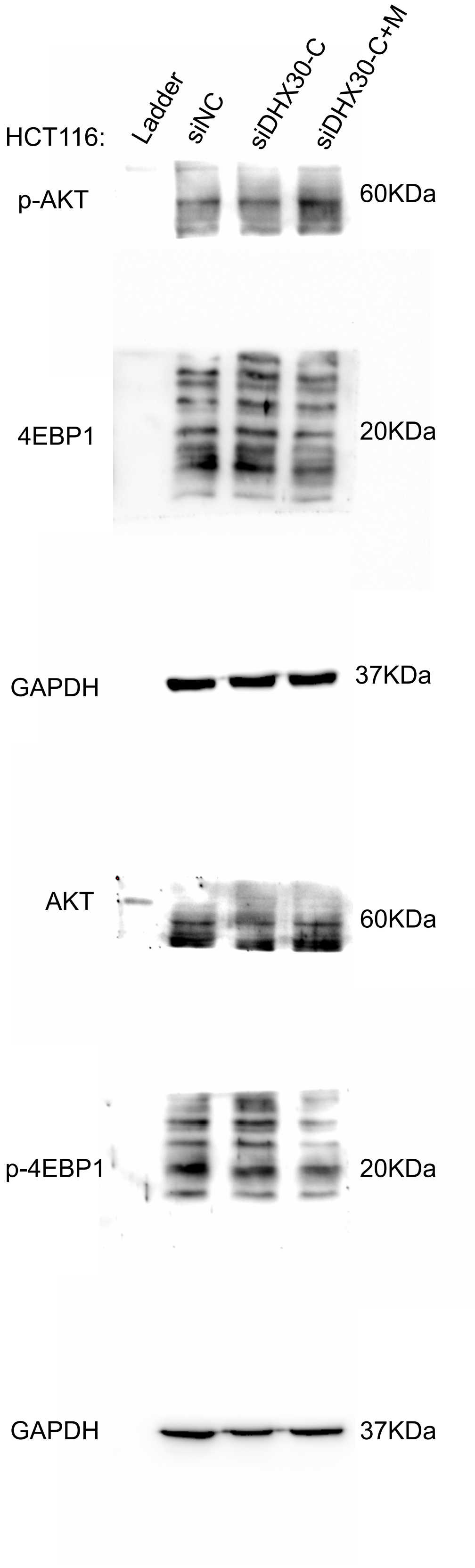
HCT116:
Ladder
siNC
siDHX30-C
siDHX30-C+M
p-AKT
60KDa
4EBP1
20KDa
GAPDH
37KDa
AKT
60KDa
p-4EBP1
20KDa
GAPDH
37KDa

Figure S3B

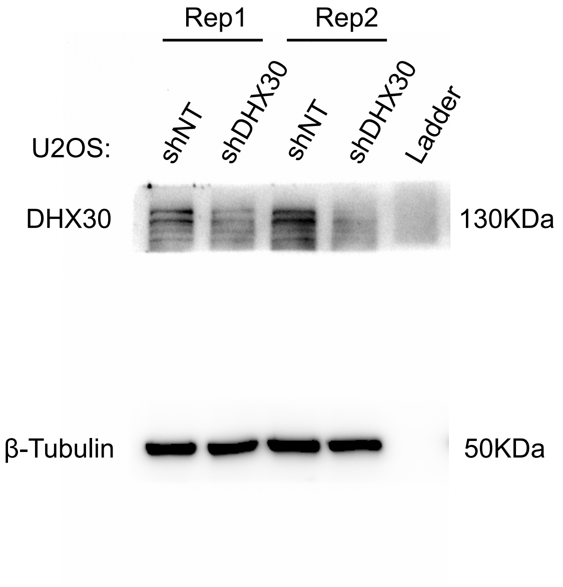

Figure S3D

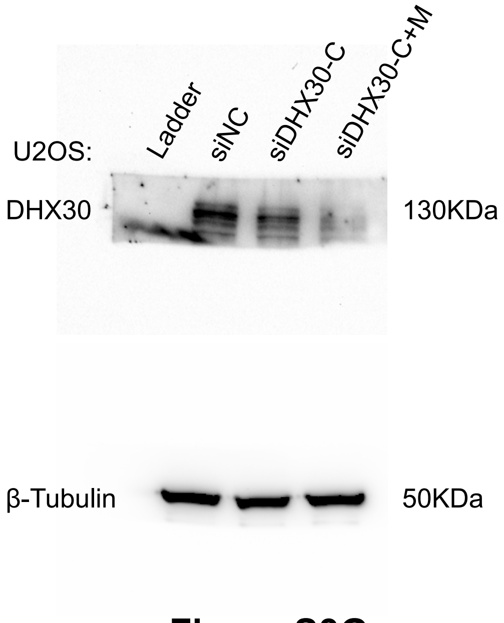

Figure S3G

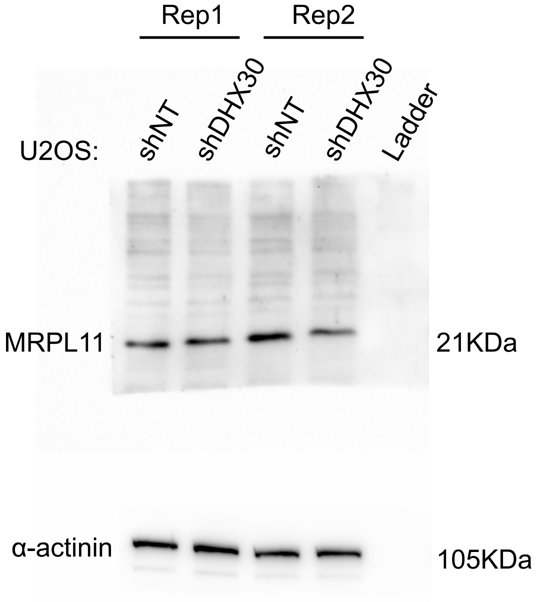

Figure S3H

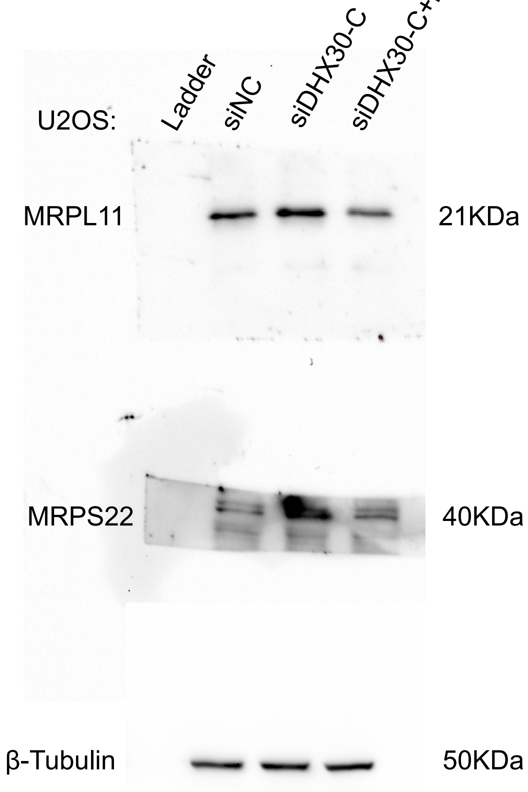

Figure S3J

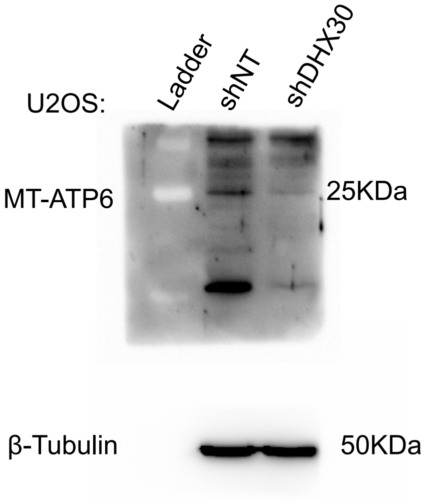

Figure S3K

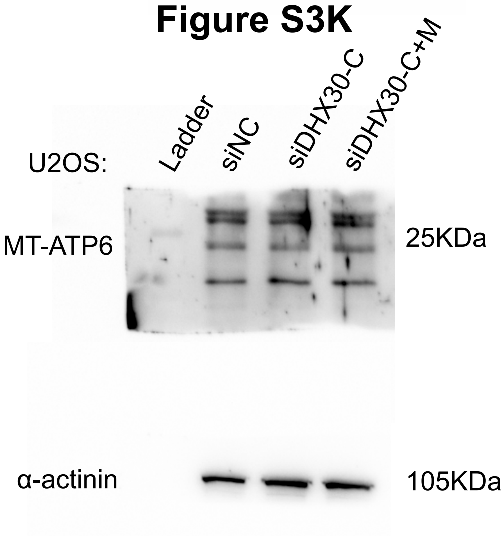

Supplement: Supplementary file 1 [file cancers-13-04412-s001.zip › Bosco et al revised Supplemental folder/uncropped WB.pdf]
