# Supplementary material for: DHX30 Coordinates Cytoplasmic Translation and Mitochondrial Function Contributing to Cancer Cell Survival
Source: Cancers (Basel). 2021 Aug 31;13(17):4412. doi: 10.3390/cancers13174412 (PMC8430983; doi:10.3390/cancers13174412)
Supplement: Supplementary file 1 [file cancers-13-04412-s001.zip › Bosco et al revised Supplemental folder/Bosco et al revised Supplemental Aug 2021.pdf]

cancers

MDPI

Supplementary Materials

DHX30 coordinates cytoplasmic translation and mitochondrial function contributing to cancer cell survival

Bartolomeo Bosco[1], Annalisa Rossi[1], Dario Rizzotto[1^], Meriem Hadjer Hamadou[1], Alessandra Bisio[1] Sebastiano Giorgetta[1], Alicia Perzolli[1], Francesco Bonollo[1], Angeline Gaucherot[2], Frédéric Catez[2], Jean-Jacques Diaz[2], Erik Dassi[1#], Alberto Inga[1#]

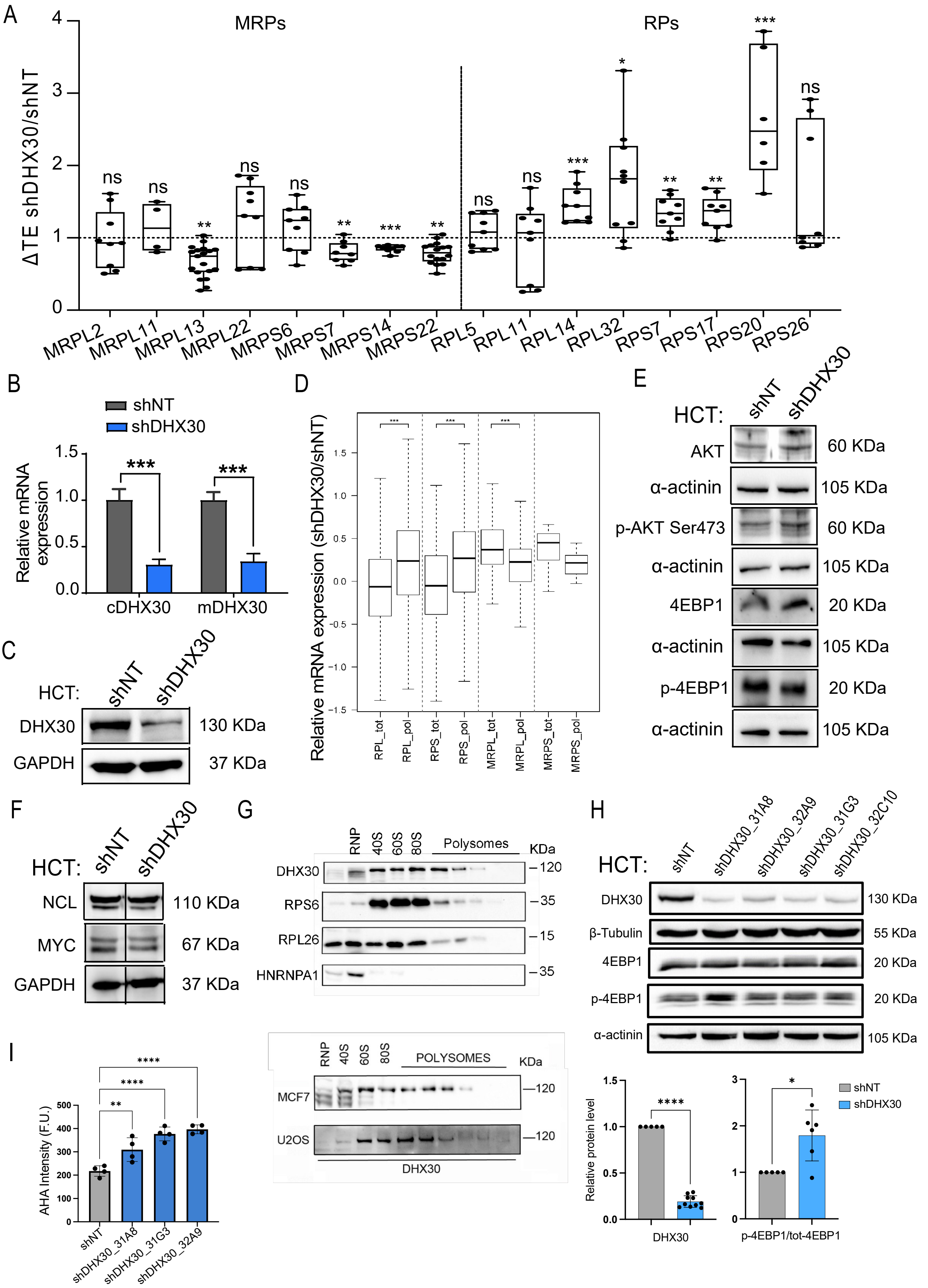

Figure S1. Relative expression of ribosomal and mitoribosomal protein transcripts, efficacy of DHX30 shRNAs, status of

mTOR and MYC pathways, association of DHX30 protein with ribosomes and polysomes, and analysis of global translation. Related to Figure 1.

(a) Relative translation efficiency obtained from RT-qPCR data for the indicated transcripts starting from total or polysomal RNA of HCT116_shNT and _shDHX30 cells. Shown is a box plot with median, minimum, maximum, and all individual data points from at least three biological replicates. YWHAZ and B2M were used as reference genes and the relative fold changes were used to compare polysomal and total RNA (Translation Efficiency = polysomal/total mRNA fold change) for HCT116_shDHX30 over _shNT. **p < 0.01; ***p < 0.001, t-test. (**b)** Relative mRNA levels of cytoplasmic (cDHX30) and mitochondrial (mDHX30) DHX30 transcripts in HCT116_shDHX30 compared to the shNT control clone (set to 1). Data are mean ± SD (n=3); ***p < 0.001, t-test. (**c**) Relative DHX30 protein expression in a representative western blot based on total proteins extracted from HCT116_shNT and _shDHX30 cells. GAPDH was used as a loading control. (**d)** DHX30 depletion leads to slight changes in the expression of ribosomal protein genes. Box plot of the expression fold changes in both total and polysomal RNAs for the indicated transcript groups in HCT_shDHX30 relative to the HCT116_shNT control. ***p < 0.001, t-test. (**e**) Immunoblot of markers fo the MTOR pathway from HCT116 shDHX30 cells (shRNA 28). Alpha-actinin was used as loading control for each gel. DHX30 protein is shown in (C). (**f)** Stable DHX30 silencing does not lead to alteration in the expression of c-MYC or of its target Nucleolin (NCL). A representative western blot image is shown. (**g**) Top panels, western blot to visualize DHX30 distribution in the different fractions of HC116 cells after polysome profiling obtained by a linear 15-50% sucrose gradient. RPS6, RPL26, and HNRNPA1 were used as controls. Bottom panels, DHX30 protein is associated with ribosomal subunits and polysomes in MCF7 and U2OS cells. Proteins were extracted from each fraction and subjected to western blot analysis using the anti-DHX30 antibody for immunodetection. (**h**) (Top) Representative western blot image of HCT116 shDHX30 clones (shRNA 31 & 32). (Bottom) Relative protein levels for DHX30 and phopsho-4EBP1 from HCT116 shNT and shDHX30 clones. Beta-tubulin and alpha-actinin intensities were used as normalization controls. Mean ± SD and individual values are shown; *p<0.05; ****p < 0.0001, one-way ANOVA. (**i**) Analysis of global translation based on the fluorescence intensity of L-azidohomoalanine (AHA) incorporated in nascent proteins present in the cytoplasm. The indicated HCT116_shDHX30 clones were compared to the shNT control in untreated condition. 5-Fluorouracil (100 µM for 16 hours) was used as control and led to about 70% signal reduction (data not shown). Mean ± SD and individual values (n=4 to 7) are shown; **p < 0.01; ****p < 0.0001, t-test.

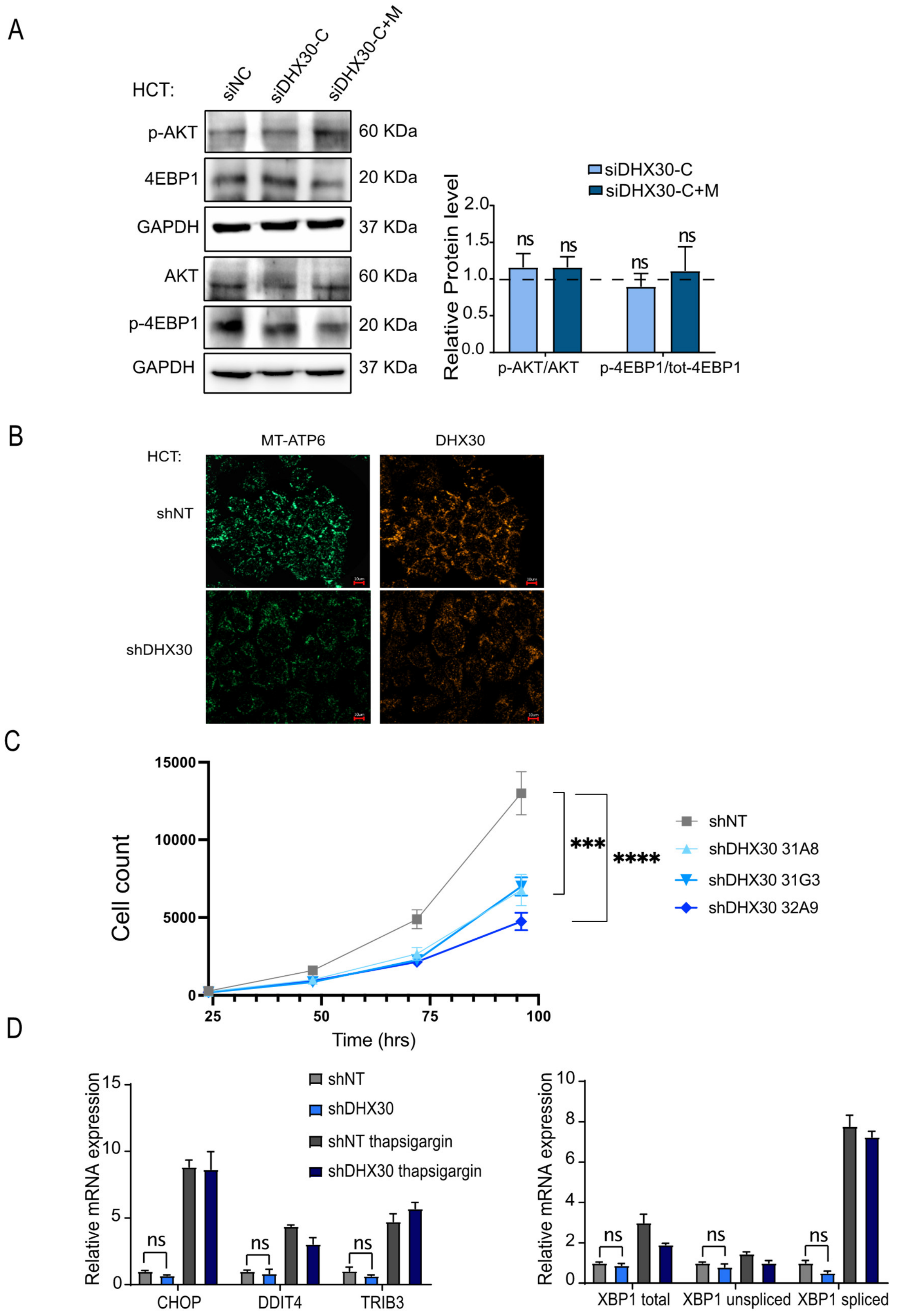

Figure S2. **Impact of transient or stable DHX30 silencing in HCT116 on the activation of the MTOR pathway, or on the expression of nuclear encoded mitoribosome components cell proliferation, or the expression of ER-stress response genes. Related to Figures 4-6.**

(a) Transient, isoform-specific silencing of DHX30 is not associated with significant activation of the mTOR pathway, based on the relative phosphorylation of AKT and 4EBP1. GAPDH was used as loading control for each gel. Representative blot images (left panel), and relative quantification of the ratio between phospho- and total- AKT or 4EBP1 and in the comparison between DHX30 silenced cells and control (siNC) cells, respectively (right panel); data are mean ± SD (n=3), t-test. The specific siRNAs used are indicated. A representative image of DHX30 protein is shown in Figure 3B. (**b**) Representative images of one of three independent immunofluorescence experiments to visualize MT-ATP6 (green) or DHX30 expression in HCT116_shDHX30 and HCT116_shNT. (**c**) Relative cell proliferation measured by high-content microscopy in digital phase contrast for the HCT116 shNT and three shDHX30 stable depleted clones (shRNA 31 and 32). For each time point and clone data are mean ± SD (n=6); ***p < 0.001; ****p < 0.0001, two-way ANOVA. (**d**) (Left panel) RT-qPCR data for the indicated ER-stress response genes. Bars plot the average fold change and the SD of three replicates. No significant differences were observed in the comparison between HCT116_shNT and _shDHX30 cells. The treatment with thapsigargin (100 nM for 24h) was included as a positive control for ER-stress. (Right panel) RT-qPCR data is used to evaluate changes in the alternative splicing pattern of the XBP1 transcript, induced downstream of an ER-stress response. There was no significant change in XBP1 splicing associated specifically with the DHX30 depletion in control, or thapsigargin treated cells. Data are mean ± SD (n=3), t-test

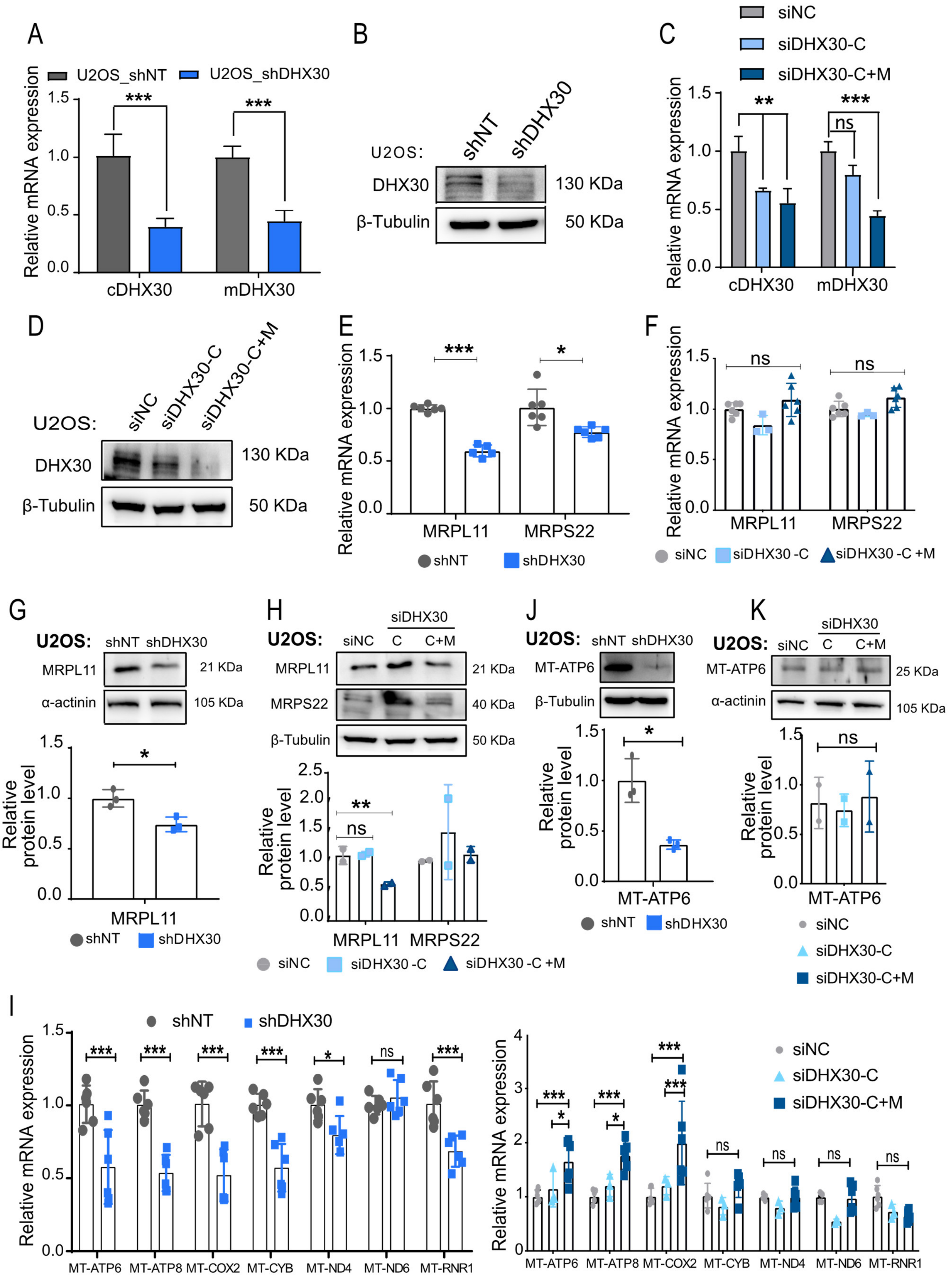

Figure S3. Impact of transient or stable DHX30 silencing on U2OS cells on mitoribosome protein transcripts or on mitochondrially encoded transcripts.

(**a**) Relative mRNA levels of cytoplasmic (cDHX30) and mitochondrial (mDHX30) DHX30 transcripts in U2OS_shDHX30 compared to the _shNT control clone (set to 1). Data are mean ± SD (n=3); ***$p < 0.001$, t-test. (**b**) Representative western blot confirming the reduction of DHX30 expression also at protein level. Beta tubulin was used as a loading control. (**c**) RT-qPCR to verify the efficacy and specificity of transient DHX30 silencing in U2OS cells. RNA was extracted 96 hours after the transfection with the indicated siRNAs. Data are mean ± SD (n=3); **$p < 0.01$; ***$p < 0.001$, one-way ANOVA. (**d**) Representative western blot image confirming the reduction of DHX30 also at protein level. Given that mDHX30 transcript is more abundant, the impact of siDHX30-C is less evident. Beta tubulin was used as a loading control. (**e**) RT-qPCR of MRPL11 and MRPS22 in U2OS_shDHX30 cells relative to the U2OS_shNT control (set to 1). Average, standard deviation, and individual data points are shown; *$p < 0.05$; ***$p < 0.001$, t-test. (**f)** RT-qPCR of MRPL11 and MRPS22 in U2OS cells transiently silenced for cytoplasmic DHX30 (siDHX30-C) or for both cytoplasmic and mitochondrial variants (siDHX30-C+M) for 96 hours. Data are compared to the siRNA negative control (siNC) and are mean ± SD (n=3); individual data points are also shown, one-way ANOVA. (**g) (**Top panel) Representative western blot of MRPL11 in U2OS_shNT or U2OS_shDHX30 cells. (Bottom panel) Relative protein quantification; mean, SD, and individual points are shown; *$p < 0.05$, t-test. (**h) (**Top panel) Representative western blot of MRPL11 and MRPS22 in U2OS transiently silenced as in (B). (Bottom panel) Relative protein quantifications; mean, confidence interval, and individual points are shown; **$p < 0.01$, one-way ANOVA. (**i**) (Left panel) RT-qPCR of selected mitochondria-encoded genes in U2OS_shDHX30 relative to the U2OS_shNT control (set to 1). (Right panel) U2OS transiently silenced for cytoplasmic DHX30 (siDHX30-C) or for both cytoplasmic and mitochondrial variants (siDHX30-C+M) for 96 hours. Data are relative to the siRNA negative control (siNC). For both panels, average, standard deviations, and individual data points are shown; *$p < 0.05$; ***$p < 0.001$, t-test. For transient silencing, data are compared to the siRNA negative control (siNC). (**j**) (Top panel) Representative western blot of MT-ATP6 and beta tubulin. (Bottom panel) Relative protein quantification of MT-ATP6 in U2OS_shNT or _shDHX30 cells. Mean, SD, and individual points are shown; *$p < 0.05$, t-test. (**k**) Same as J but for transiently silenced U2OS cells. Mean, confidence interval, and individual points (two biological replicates) are shown, one-way ANOVA.

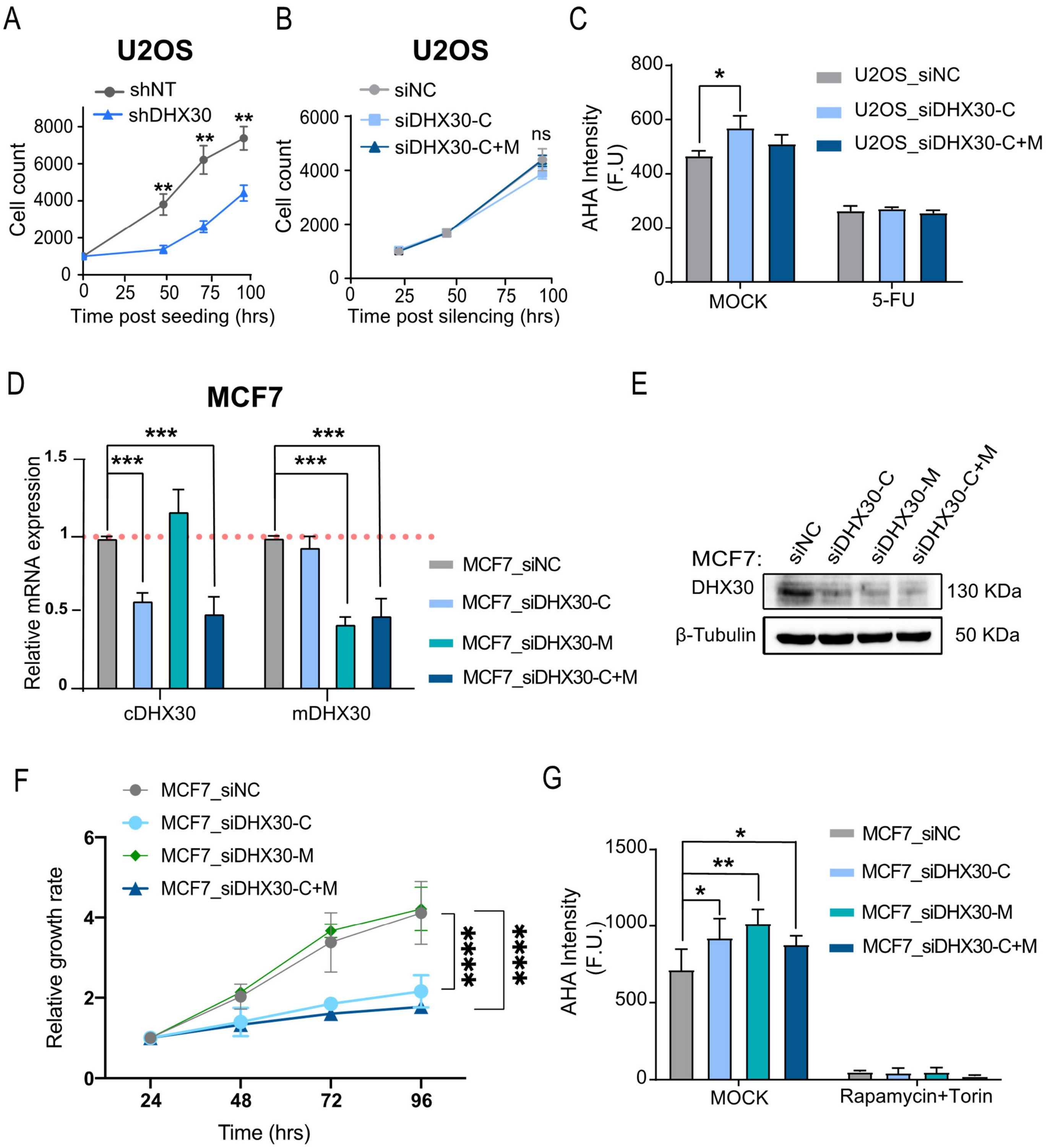

Figure S4. Impact of transient, isoform specific DHX30 silencing on cell proliferation and global translation in U2OS and MCF7 cells.

(**a**) Relative cell proliferation measured by high-content microscopy in digital phase contrast in U2OS_shNT or U2OS_shDHX30 cells. **p < 0.001, two-way ANOVA. (**b**) U2OS cells were transiently silenced using the indicated siRNAs and proliferation was monitored as in A, starting twenty-four hours after transfection, two-way ANOVA. (**c**) Analysis of global translation based on L-azidohomoalanine (AHA) fluorescence intensity measured from cytoplasmic proteins in U2OS cells transiently transfected as indicated, for 96 hours. 5-Fluorouracil treatment was used as a control leading to reduced translation. Data are mean ± SD (n=3); *p

< 0.05, one-way ANOVA. (**d**) RT-qPCR to verify the efficacy and specificity of transient DHX30 silencing in MCF7 cells. RNA was extracted 96 hours after the transfection with the indicated siRNAs. Data are mean ± SD (n=3); ***$p < 0.001$, one-way ANOVA. (**e**) Representative western blot image confirming the reduction of DHX30 protein level. Given that mDHX30 is more abundant, the impact of siDHX30-C on protein level is slightly less evident. (**f**) Relative cell proliferation measured in transiently silenced MCF7 cells by high-content microscopy in digital phase contrast, as done for Figure 6G. Cells were counted starting 24 hours after transfection and every 24 hours till the 96hr time point. Data is plotted relative to the 24-hour time point. n=3, **** $p<0.0001$ for both the 72- and 96-hour time point, two-way ANOVA. (**g**) Analysis of global translation based on L-azidohomoalanine (AHA) fluorescence intensity measured from cytoplasmic proteins in MCF7 cells transiently transfected as indicated, for 96 hours. Data are mean ± SD (n=3) *$p < 0.05$; **$p < 0.001$, one-way ANOVA. Rapamycin plus Torin treatment for 16 hours was used as a control inhibiting translation initiation, as MCF7 cells were more resistant to 5-FU compared to HCT116 cells.

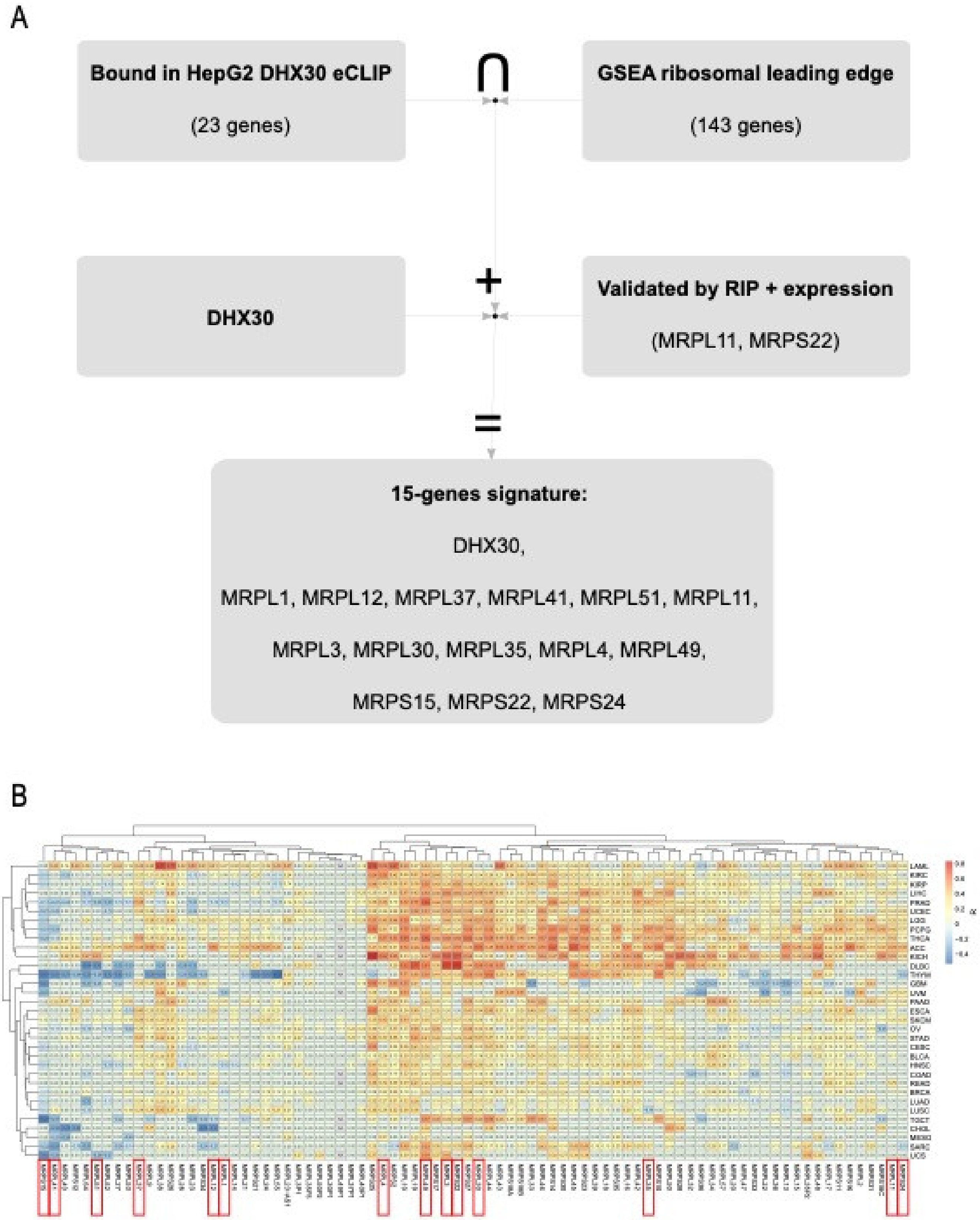
A
Bound in HepG2 DHX30 eCLIP
(23 genes)
∩
GSEA ribosomal leading edge
(143 genes)
DHX30
+
Validated by RIP + expression
(MRPL11, MRPS22)
=
15-genes signature:
DHX30,
MRPL1, MRPL12, MRPL37, MRPL41, MRPL51, MRPL11,
MRPL3, MRPL30, MRPL35, MRPL4, MRPL49,
MRPS15, MRPS22, MRPS24
B

C

		Data extracted from the GEPIA2 web server^							
		Expression Correlation*		Outcome					
clustering	tumor type	R	p-value	HR (high)	Logrank p value	p(HR)	n	tumor type name
Cluster1	LAML	0.5	3.10E-12	**2.4**	**0.016**	**0.02**	54	Acute Myeloid Leukemia
	KIRC	0.39	0	**0.69**	**0.017**	**0.017**	516	Kidney renal clear cell carcinoma
	KIRP	0.44	8.90E-15	0.87	0.65	0.65	282	Kidney renal papillary cell carcinoma
	LIHC	0.49	0	**1.9**	**0.00027**	**0.00035**	364	Liver hepatocellular carcinoma
	PRAD	0.44	0	***2.1***	***0.00063***	***0.00083***	492	Prostate adenocarcinoma
	UCEC	0.4	5.70E-11	0.64	0.21	0.22	172	Uterine Corpus Endometrial Carcinoma
	LGG	0.42	0	**1.7**	**0.0045**	**0.005**	514	Brain Lower Grade Glioma
	PCPG	0.52	3.60E-14	*1.2*	*0.74*	*0.74*	182	Pheochromocytoma and Paraganglioma
	THCA	0.59	0	*0.66*	*0.16*	*0.16*	510	Thyroid carcinoma
	ACC	0.67	2.40E-11	**3.3**	**0.0035**	**0.0055**	76	Adrenocortical carcinoma
	KICH	0.66	1.70E-09	**9.5**	**0.0096**	**0.0034**	64	Kidney Chromophobe
Cluster 2	DLBC	0.37	1.00E-02	0.65	0.56	0.56	46	Lymphoid Neoplasm Diffuse Large B-cell Lymphoma
	THYM	0.21	2.20E-02	*1.5*	*0.37*	*0.37*	119	Thymoma
	GBM	0.13	9.30E-02	0.98	0.86	0.9	162	Glioblastoma multiforme
	UVM	0.04	7.30E-01	**3**	**0.025**	**0.033**	78	Uveal Melanoma
	PAAD	0.41	1.70E-08	1.3	0.21	0.22	178	Pancreatic adenocarcinoma
	ESCA	0.31	2.30E-05	1	0.99	1	182	Esophageal carcinoma
	SKCM	0.44	0.00E+00	**1.4**	**0.027**	**0.028**	458	Skin Cutaneous Melanoma
	OV	0.17	3.70E-04	0.85	0.18	0.19	424	Ovarian serous cystadenocarcinoma
	STAD	0.35	3.00E-13	0.82	0.21	0.22	384	Stomach adenocarcinoma
	CESC	0.21	3.10E-04	1.1	0.75	0.75	292	Cervical squamous cell carcinoma and endocervical adenocarcinoma
	BLCA	0.31	2.80E-10	1.1	0.63	0.63	402	Bladder Urothelial Carcinoma
	HNSC	0.22	2.70E-07	1.1	0.32	0.33	518	Head and Neck squamous cell carcinoma
	COAD	0.17	4.90E-03	0.79	0.33	0.34	270	Colon adenocarcinoma
	READ	0.37	2.80E-04	0.74	0.53	0.53	92	Rectum adenocarcinoma
	BRCA	0.22	1.70E-13	1.3	0.13	0.13	1070	Breast invasive carcinoma
	LUAD	0.16	6.10E-04	**1.6**	**0.0027**	**0.003**	478	Lung adenocarcinoma
	LUSC	0.2	6.80E-06	0.86	0.27	0.27	482	Lung squamous cell carcinoma
	TGCT	0.17	5.40E-02	*1.4*	*0.34*	*0.34*	136	Testicular Germ Cell Tumors
	CHOL	-0.16	3.70E-01	1.1	0.79	0.8	36	Cholangio carcinoma
	MESO	0.05	6.60E-01	1.5	0.097	0.098	82	Mesothelioma
	SARC	0.12	6.20E-02	1.3	0.25	0.25	262	Sarcoma
	UCS	0.03	8.50E-01	1.2	0.54	0.53	56	Uterine Carcinosarcoma

^ http://gepia2.cancer-pku.cn/#index

* between DXH30 and 14 mitoribosomal transcripts that are derived from the intersection of Translation Efficiency data, Leading Edge in significant terms in GSEA and DH30 eCLIP data in ENCODE: MRPL1, MRPL12, MRPL3, MRPL30, MRPL35, MRPL37, MRPL4, MRPL41, MRPL49, MRPL51, MRPS15, MRPS24, MRPS22, MRPL11

n refers to the total number of samples. These were split at median for the K-M analysis.

Number in italics indicate the DFS instead of OS data was used.

D

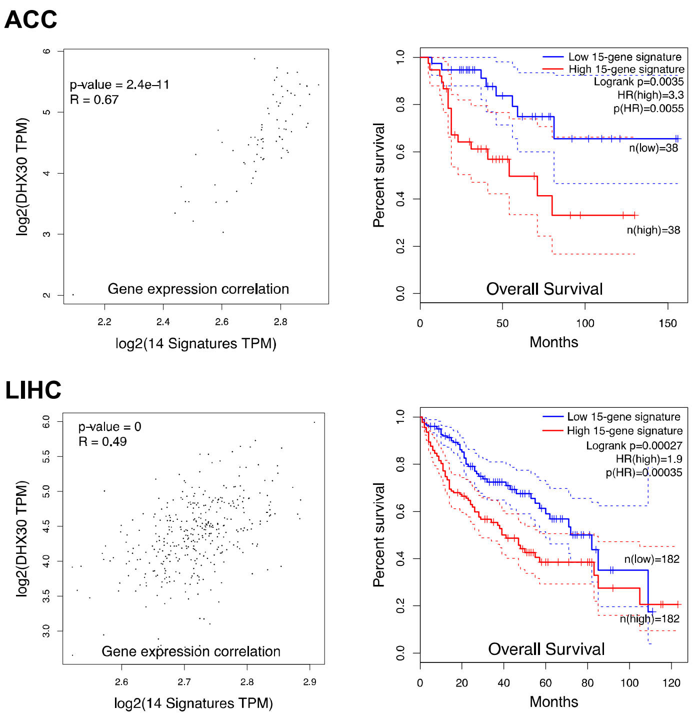

Figure S5. DHX30 expression positively correlates with the expression of mitoribosomal protein transcripts and could have prognostic significance.

(**a**) Workflow that led to a 15-gene signature comprising DHX30 and 14 mitoribosomal gene transcripts. ENCODE eCLIP data obtained in HEPG2 were cross-referenced with information from the GSEA analysis of differentially expressed genes from our RNA-seq data of HCT116_shDHX30 cells and integrated with results from RIP and gene expression studies performed in this work. (**b**) Pearson correlation between the expression of DHX30 and each mitoribosomal protein transcript in cancer samples from TGCA. Data

were extracted from the GEPIA web server (see methods for details). Unsupervised clustering revealed two major clusters based on the level of gene expression correlation and two main clusters among cancer types. Boxed in red are the names of the fourteen transcripts that are candidate DHX30 direct targets (see text for details). (**c**) The table presents the expression correlation data (R value and p value) between DHX30 and the combined group of 14 mitoribosomal protein transcripts is listed for each cancer type). Data from Kaplan-Meier survival analysis for the aggregated 15-gene signature (DHX30 + the 14 mitoribosomal protein transcripts) is also shown. Significant differences are highlighted in bold font. The tumor type acronym and extended name are given. (**d**) The graphs present two examples among the cluster of eleven cancer types where a positive correlation is apparent. (Left panels) Pearson correlation. (Right panels) Overall Survival Kaplan-Meier. The correlation value, the Hazardous Ratio, p values and sample sizes are shown.

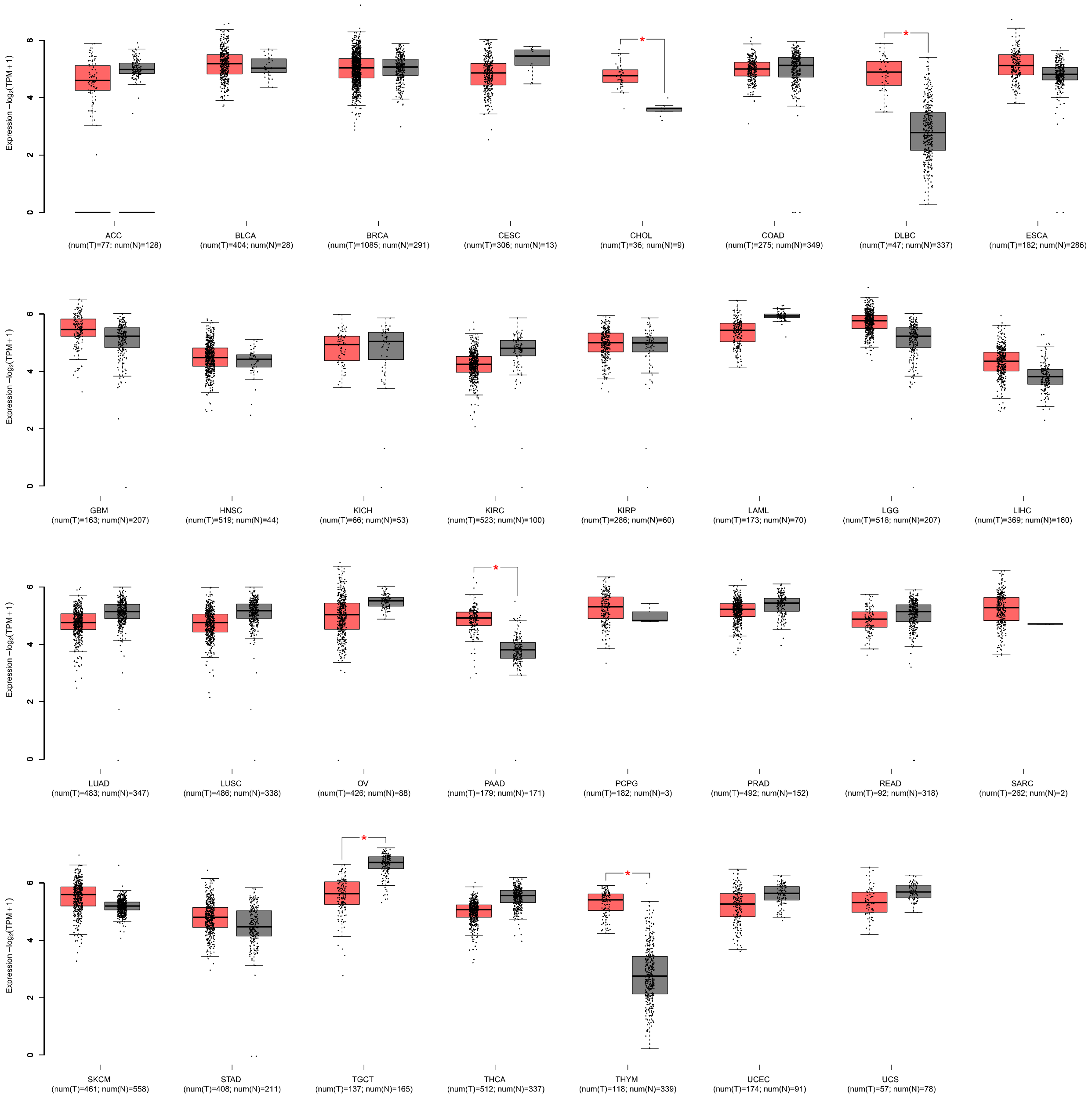

Figure S6. Expression of DHX30 in TCGA tumor types and matched healthy tissues.

Each boxplot pair displays the expression levels of DHX30 in a TCGA tumor samples (red box) and in the corresponding healthy tissue samples (grey box, composed of TCGA normal samples and GTEX samples of the same tissue). Expression is displayed in log2(tags per million sequenced reads + 1). Plots were obtained with GEPIA2. Significance of the difference between tumor and normal samples is indicated by a red asterisk and represents a q-value< = 0.01 and a |log2FC|>1 as computed in GEPIA2 by the ANOVA method.

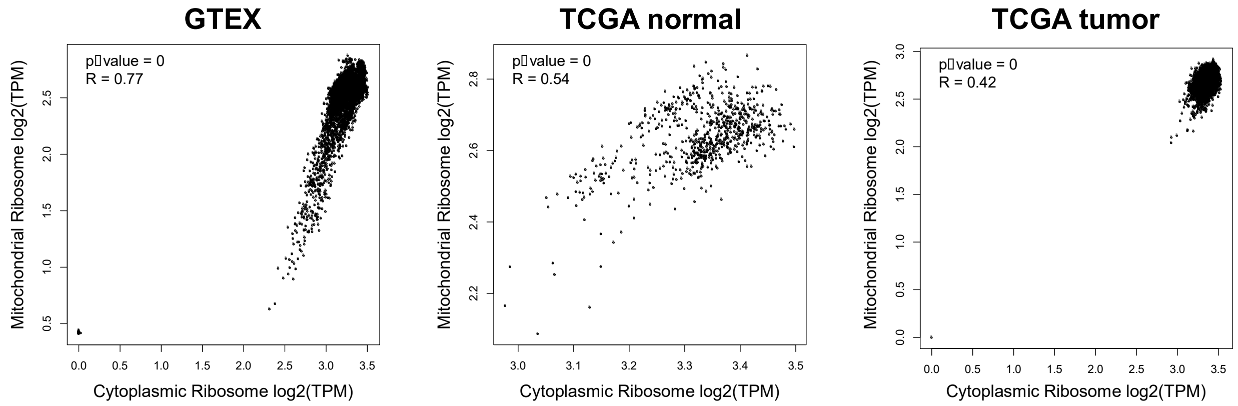

Figure S7. Expression correlation of cytoplasmic and mitochondrial ribosome component genes in TCGA and GTEX samples. The three scatterplots display the Pearson correlation (R and p-value indicated in the upper left corner of each plot) between the expression (indicated as log2(tags per million sequenced reads)) of cytoplasmic ribosome component genes (X axis) and mitochondrial ribosome component genes (Y axis). The leftmost plot shows the correlation in GTEX healthy tissues samples, the center plot presents the correlation in TCGA normal samples, and the rightmost plot shows the correlation in TCGA tumor samples. Plots were obtained with GEPIA2.
